# Climate Extremes, Genomic Coverage, and Taxonomy Shape the Detection of Adaptation: A Systematic Review of GEA Studies for the Kingdom Animalia

**DOI:** 10.1111/mec.70328

**Published:** 2026-03-25

**Authors:** Allison K. Williams, Olivia M. Ruppert, Erin L. Landguth

**Affiliations:** ^1^ School of Environment & Natural Resources, College of Food, Agriculture, and Environmental Sciences The Ohio State University Columbus Ohio USA; ^2^ School of Public and Community Health Sciences University of Montana Missoula Montana USA

**Keywords:** adaptive variation, animalia, environment, single nucleotide polymorphism

## Abstract

Genotype–environment association (GEA) is widely used for identifying genetic variation linked to environmental pressures. Their proliferation over the past decade offers a critical opportunity to synthesize how adaptive variation is identified and distributed across broad taxonomic groups. Here, we reviewed 194 GEA studies from the Kingdom Animalia to summarize the analytical methods employed and the key predictors of adaptive variation. Our review revealed that latent factor mixed models (LFMM) and redundancy analyses (RDA) are the most frequently used methods, with most studies employing multiple analytical approaches. On average, studies sampled approximately 0.05% of the focal species' genome (SD = 0.14%). Across studies using genome‐representative markers, we identified a non‐linear relationship between genome coverage and candidate loci detected, indicating diminishing returns beyond 0.45% genomic coverage, supporting the importance of factors beyond dataset size. Climatic variables reflecting extremes and variation were most consistent for detecting candidate loci, but these patterns varied greatly across taxa. We also identified influential taxon‐specific environmental relationships for bony fishes, arthropods, mammals, birds, herpetofauna, and molluscs and highlighted influential variables to inform future research efforts. This synthesis confirms the tight linkage between adaptive variation and species' ecology, offering a quantitative guide for future study design to improve statistical detection power, and prioritize the environmental drivers shaping evolution.

## 1 Introduction

Assessing adaptive variation using genomic data is a cornerstone of modern evolutionary biology (Hoban et al. 2016). Methods to identify adaptive variation include genome‐wide association studies (GWAS, e.g., PLINK and GEMMA), outlier tests (e.g., Bayescan, Arlequin and PCAdapt) and genotype‐environment association (GEA) analyses (Forester, Lasky, et al. 2018; Lasky et al. 2023; Lotterhos and Whitlock 2014). GWAS and outlier tests identify loci that may be under selection by identifying statistical associations between genetic variants and phenotypic traits or by flagging loci with unusually high patterns of genetic differentiation. In contrast, GEA analyses directly assess the relationship between genetic variation and environmental gradients, providing insight into the selective pressures that may be shaping spatial patterns of adaptation (Forester, Landguth, et al. 2018). In this context, GEA patterns reveal fundamental aspects of species' biology, as well as landscape‐level processes such as gene flow, population persistence across environmental gradients, and local adaptive potential (Flanagan et al. 2018). This information can also be used for applied management and conservation, especially in the face of increasing environmental pressures such as habitat fragmentation, biodiversity loss, and climate change (Forester et al. 2025; Forester, Landguth, et al. 2018; Razgour et al. 2018). As a result of these applications, GEA analyses have become the most common landscape genetic approach to understanding adaptive variation across environmental gradients (Balkenhol et al. 2017; Forester, Landguth, et al. 2018; Rellstab et al. 2015).

While GEA approaches have become increasingly common, many methodological uncertainties remain. For example, correcting for population structure can reduce power to detect true adaptive signals, while ignoring structure can result in the false‐positive identification of outliers (Forester, Lasky, et al. 2018; Rellstab et al. 2015). Furthermore, it can be challenging to estimate how much genomic coverage is needed to reliably detect adaptive loci, and this can create uncertainty about potential false negative results. Also, studies may fail to detect adaptive loci if the chosen environmental predictors do not align with species ecology (Hoban et al. 2016). These uncertainties contribute to confusion about whether studies that fail to find adaptive loci are truly negative or are underpowered by their choice of markers and environmental predictors.

The combination of this massively growing field and these continuous uncertainties motivated us to conduct a systematic review of GEA studies. We intentionally limited this goal to the kingdom Animalia, excluding others (Archaea, Bacteria, Protista, Fungi and Plantae). Although GEA approaches were developed early and have been widely applied in other systems such as plants, our aim was to synthesize and evaluate how these methods have been adopted and adapted for animal systems specifically, in which biological characteristics such as high mobility, broad or shifting home ranges, and complex demographic histories can complicate genotype–environment associations. Our first objective was to review the methods used to conduct GEA analyses, with the purpose of describing the current body of literature and highlighting effective methods for identifying candidate loci. To accomplish this, we collected data on method(s) employed, modelling choices made (e.g., corrections for structure), marker origin and genomic coverage across studies. Second, we aimed to summarize the findings across GEA studies, such as the significant environmental predictors used, the number of outlier loci detected, and the taxonomic groups that were studied. We also explored predictor variables by categorizing them in multiple ways and compared their relationships across taxonomic groups to identify taxa‐specific trends.

By examining how GEA analyses are applied and how their findings vary across taxa, we identified broader patterns in methodological choices, environmental predictors, and the detection of adaptive genetic variation. These patterns provide insight into both the evolutionary processes shaping local adaptation and the practical considerations involved in applying GEA to real‐world conservation challenges. Ultimately, this review highlights how GEA analyses have been conducted, identifies environmental factors influencing local adaptation, and outlines how future research can build on current trends to better understand and conserve biodiversity in a changing environment.

## 2 Materials and Methods

This systematic review was based on Preferred Reporting Items for Systematic Reviews and Meta‐Analyses (PRISMA; Page et al. 2021, see File S1 for the PRISMA flow diagram and checklist).

### 2.1 Data Sources and Search Strategy

Our literature search included two primary databases: Web of Science Core Collection (WoS) and Google Scholar. To ensure the relevance of studies, we used the search phrase: ‘genotype–environment association*’. This narrow phrase appropriately captured wildlife genotype–environment association (GEA) studies while avoiding large numbers of human research studies (e.g., genotype–environment interaction studies). However, reliance on this strict terminology may have excluded studies using alternative phrasing (e.g., ‘genetic–environment association’), and some relevant studies may therefore not have been captured.

### 2.2 Study Selection, Eligibility Criteria and Quality Assessment

We considered studies published in English up to January 2025. Eligible studies were primary research (including peer‐reviewed publications, pre‐prints, theses and dissertations) that conducted GEAs on non‐human species in the kingdom Animalia. We excluded studies on non‐Animalia species, human subjects, non‐English texts, non‐GEA analyses, and studies focused solely on methods development. A PRISMA flow diagram illustrates the study selection process (File S1). Two reviewers (A.K.W. and O.M.R.) independently screened titles, abstracts, and full texts and systematically extracted relevant information from each eligible study based on the pre‐defined forms (File S2). Discrepancies regarding study eligibility, such as ‘borderline’ studies with insufficient methodological detail, were resolved through consensus between reviewers. This ensured consistent and transparent application of inclusion criteria to all screened studies.

### 2.3 Summary of Measures and Synthesis

#### 2.3.1 Objective 1: Review Methods Used in GEA Analyses

We used R (version 4.4.2; R Core Team 2024) and RStudio (version 2024.12.0; RStudio Team 2024) with the package ‘tidyverse’ (Wickham et al. 2019) to filter, sort, and visualize results. Each GEA analysis within a study was treated as an independent observation, although multiple analyses were often conducted on the same taxa or populations within a study. For each analysis, we recorded the GEA method, marker origin and number, use and type of genetic (e.g., latent factors and covariance matrices) and/or geographic structure (e.g., coordinates or inclusion of Moran's Eigenvector Maps as variables) correction, number of outliers detected, use of multiple methods and shared loci, dimensionality reduction and validation approach.

To inform future study design, we examined the relationship between genomic sampling effort and the detection of putatively adaptive loci across studies. Genomic sampling effort was quantified as genomic coverage, calculated from reported marker counts divided by the published genome size estimates for the focal species or closely related taxa (see genome size sources in File S2). For this analysis alone, we omitted studies using non‐representative (i.e., targeted) and transcriptomic marker origins (File S3). We then evaluated how the number of detected outliers varied with increasing genomic coverage by fitting both linear and non‐linear models to the observed data. Model fit was compared using a likelihood ratio test and Akaike's Information Criterion. These models summarize empirical patterns in outlier detection and are not intended to estimate the true number of adaptive loci.

#### 2.3.2 Objective 2: Summary of Environmental Predictors Retained and Taxonomic Coverage Across GEA Analyses

We recorded all environmental variables retained in each GEA analysis, the justification provided for their inclusion, and the number of candidate loci significantly associated with each variable, when reported. We then categorized each variable at three nested scales: (1) finest scale (the finest comparable categorization for individual variables), (2) variable type (e.g., temperature and precipitation), and (3) measurement type (e.g., mean, extreme and raw). To quantify the effectiveness of different variable categories, we calculated a ‘SNP detection rate’ as the number of candidate outlier SNPs associated with each variable divided by the total number of SNPs used in that analysis. For comparison and visibility, we plotted the overall log‐scaled mean SNP detection rate and 95% confidence intervals for each variable category. To explore taxon‐specific trends, we recorded species, order and broader taxonomic classification for each study and calculated SNP detection rates for each group. We visualized the log‐scaled median detection rates and their distributions, excluding any taxon with less than 10 observations and variable‐taxon combination with less than five observations, and descriptively summarize these patterns.

## 3 Results

### 3.1 Literature Search and Study Selection

The initial search yielded 877 results from Google Scholar and 207 results from WoS (File S1). Across two rounds of iterative filtering (first through titles and abstracts, then through full text), 889 results were excluded with the following reasons: duplication (*n* = 121), not conducting a GEA (*n* = 113), inaccessible formatting (e.g., non‐English texts; *n* = 129), human studies (*n* = 24), species outside the kingdom Animalia (*n* = 417), focused on methods development rather than inference (*n* = 71), and incomplete reporting (*n* = 15). Incomplete reporting included studies having excessive missing information. Finally, we retained 194 studies for systematic review (File S2).

### 3.2 Methodological Trends in GEA Literature

**FIGURE 1:**
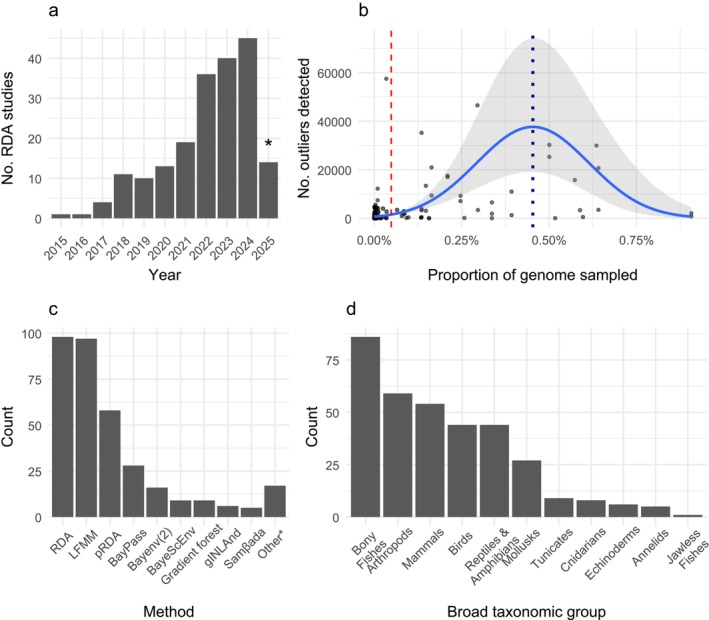
(a) Histogram of publications over time (*this systematic review only included publications until January 2025). (b) Scatterplot displaying the genomic coverage of each study versus the candidate outliers detected by each GEA analysis. A solid blue line and shading depict the mean and 95% confidence interval of the quadratic negative binomial model. A blue dotted vertical line at 0.45% notes where the slope is zero, where candidate outlier detection begins to decrease with increasing genomic coverage. The red vertical line at 0.05% notes the average genomic coverage across analyses. (c) Bar plot displaying the frequency of GEA methods used. (d) Bar plots displaying the frequency of GEA studies on broad taxonomic groups. LFMM, latent factor mixed model; pRDA, partial redundancy analysis; RDA, redundancy analysis.

The majority of GEA studies in this review were published between 2022 and 2025 (Figure 1a). Across 194 independent studies, 343 GEA analyses were conducted, with an average of 1.77 GEA analyses used per study. The most common response variables were population‐level allele frequencies and individual genotypes. Most studies used some kind of reduced‐representation sequencing (67%) followed by whole‐genome shotgun‐based approaches (23%). Targeted (7%) and transcriptomic/metagenomic approaches (2%) were much less common. Analyses included a mean of 1,072,683 SNPs (SD = 4,431,838), corresponding to an average genomic coverage (i.e., marker density/genome size) of 0.05% (SD = 0.14%) (Figure 1b, red line). An average of 1977 SNPs (SD = 6067) per study were identified as outliers, and an average of 657 (SD = 2559) were repeatedly identified across studies that used multiple GEA methods.

We observed a non‐linear relationship between genomic coverage and the number of detected outliers, which was better described by a quadratic negative binomial model than by a linear model (*p* < 0.001; Figure 1b; File S3). The fitted model indicated that gains in detected outliers increased rapidly at low genomic coverage, plateauing at approximately 0.45% (Figure 1b, blue line). Beyond this plateau, the model predicted a decline in outlier detection (File S3).

Population‐structure‐aware models (*n* = 158; LFMM, BayPass, Bayenv/Bayenv2, BayeScEnv) and ordination‐based multivariate methods (*n* = 156; RDA and pRDA) dominated GEA methods (Figure 1c, File S2; Caye et al. 2019; Frichot et al. 2013). Less common methods included machine‐learning, spatial‐autocorrelation frameworks and regression models.

Across analyses, 56% corrected for population structure, 22% for geographic structure, 9% corrected both, and 30% did not make any corrections (File S3). Genetic structure was most frequently accounted for by including latent factors or covariance matrices in population‐structure aware methods such as LFMM and BayPass (File S2). In ordination‐based approaches, authors often used conditioning variables derived from principal component, admixture or clustering analyses. Spatial structure was most often corrected using variables derived from Moran's Eigenvector Maps or by directly including coordinates as variables. When structure was not explicitly corrected for, most analyses provided no justification; when explanations were given, authors most often cited the use of multiple complementary methods, concerns about overcorrection, low inferred structure, methodological robustness to structure, sampling design that accounted for structure or assumptions that spatial corrections captured genetic structure (File S2). We did not detect any difference in SNP detection rates based on whether or not corrections were applied (File S3).

Almost all (96%) studies reported some form of validation for outlier loci. Validation methods were most commonly statistical (88%), cross‐method agreement among multiple GEA approaches (69%), and functional annotation of candidate loci (65%). Most used at least two forms (80%) and nearly half (46%) applied all three validation strategies.

### 3.3 Taxonomic Coverage and Environmental Predictors of Candidate Loci

**FIGURE 2:**
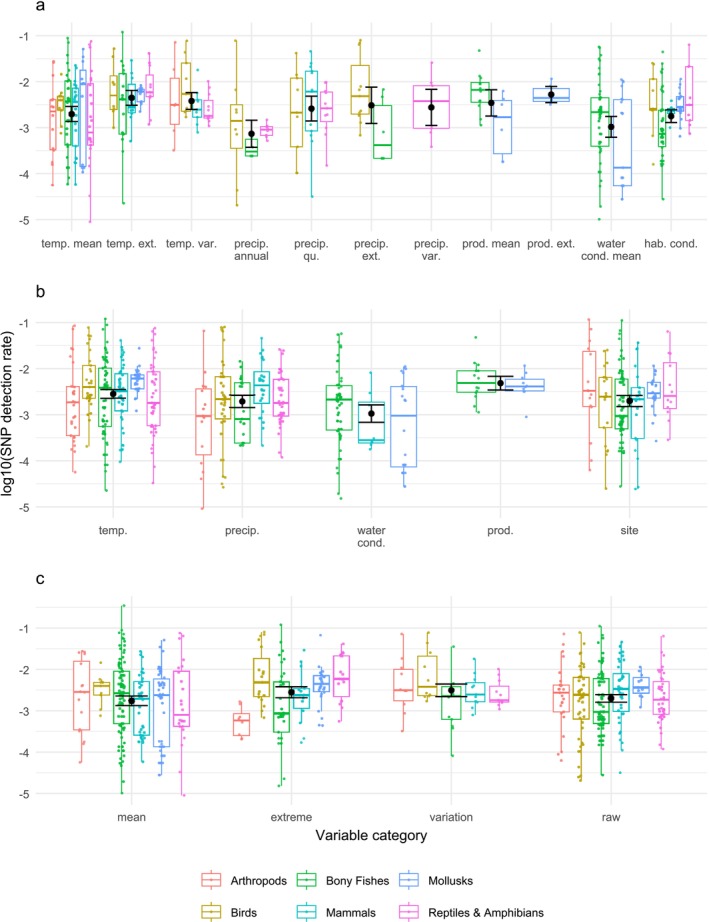
(a–c) Three sets of boxplots displaying the log‐scaled median (middle line) inner 50% quartile (box), general spread of the data (whiskers) for the SNP detection rate across different variable categories and broad taxonomic groups. (a) displays the finest scale grouping of variables where variables such as temperature and precipitation are split into mean, extreme, and variation variables, (b) displays relationships by calculation type and (c) displays relationships by variable type (see File S2 for more detail). To prevent low sample size issues, we filtered taxonomic groups with less than 10 analyses and variable‐taxon combinations with less than five observations. cond. = conditions; ext. = extreme; hab. = habitat; precip. = precipitation; prod. = productivity; qu. = quarterly; temp. = temperature; var. = variation.

Our review found that GEA studies were concentrated in several major taxonomic groups: Bony fishes, arthropods, mammals, birds, herpetofauna, and molluscs (Figure 1d, File S2). 75% of studies provided a rationale for variable selection, most often biological or mechanistic justifications (69%), followed by exploratory (4%) and literature‐based (2%) rationales. Dimension reduction using principal components analyses was used in 18% of analyses, most often in structure‐aware methods like LFMM (63%) followed by ordination‐based approaches (34%) (File S2). Since not all taxa are represented evenly within each variable category, the relationships between SNP detection rates of variables across taxa are presented descriptively rather than tested statistically (see File S2 for more detail on variable classification). We classified SNP detection rates as high when the median for a taxonomic group exceeded the overall interquartile range, moderate when it fell within the range and low when it fell below the range. Productivity variables had higher detection rates relative to other variable categories (Figure 2b). Variables measuring extremes or variability were associated with higher SNP detection rates than other measurement categories (Figure 2c).

Arthropods showed high detection rates for site and mean variables, moderate rates for temperature, variation and raw (i.e., not summarized) variables, and low rates for precipitation and extreme variables (Figure 2b,c). Birds had high detection rates for temperature, mean, and extreme variables, and moderate rates for precipitation, site, variation, and raw variables. Bony fishes had high detection rates for temperature, water conditions, and mean variables, moderate rates for productivity and variation, and low rates for precipitation, site, extreme, and raw variables. Mammals had high detection rates for precipitation and raw variables, moderate rates for temperature, site, mean, extreme and variation variables, and low rates for water conditions. Molluscs had high detection rates for temperature, site, extreme, and raw variables and moderate rates for water conditions, productivity, and mean variables. Herpetofauna had high detection rates for extremes, moderate rates for precipitation, site, variation, and raw variables, and low rates for temperature, mean, and variation variables.

## 4 Discussion

The field of GEA studies has expanded rapidly, with a surge in publications between 2022 and 2025 driven by the increasing availability of large genomic datasets. Our systematic review of nearly 200 studies provides a quantitative snapshot of this growing discipline. We found that while RDA is the most common approach, nearly half of all studies employ multiple methods to identify candidate loci. Using a quadratic negative binomial model, we found a non‐linear relationship between genomic coverage and number of outlier loci detected, with rapid gains at low coverage before plateauing at 0.45%, beyond which the trend became negative. Across all taxa, we found that climatic variables, particularly those measuring extremes and variability, were powerful predictors of adaptation. Furthermore, we identified distinct taxon‐specific relationships between environment and adaptive variation, confirming that the choice of environmental predictors strongly influences the outcomes of GEA analyses.

### 4.1 Methodological Implications

The genomic coverage required for a successful GEA study is contingent on many complex and competing factors, including the research question, species biology, sampling design, and SNP origin (Forester, Lasky, et al. 2018). The substantial variation in the observed relationship between genomic coverage and outliers detected likely reflects this complexity. Nevertheless, the quadratic model indicates that increases in genomic coverage do not necessarily translate into continuous gains in outlier detection and instead show that there may be diminishing returns at higher levels of genomic sampling. Rather, studies attempting to identify adaptive signals should balance marker density with other considerations, such as the number and spatial distribution of samples, environmental heterogeneity, and the genomic architecture of adaptive traits (Flanagan et al. 2018; Forester, Lasky, et al. 2018; Hoban et al. 2016). For instance, studies with sparse spatial sampling may require more markers to achieve the same power as a well‐sampled study with strong environmental contrasts (De Mita et al. 2013; Lotterhos and Whitlock 2014; Rellstab et al. 2015). Therefore, genomic coverage patterns should be used to inform, not dictate, study design.

Since the effectiveness of incorporating corrections into outlier detection depends on the system, sampling design, and modelling framework, the adequacy of corrections cannot be evaluated directly (Hoban et al. 2016). There is some debate in the literature over whether corrections adequately reduce false positives or overcorrect, preventing the detection of adaptive loci (Forester, Lasky, et al. 2018; Hoban et al. 2016; Rellstab et al. 2015). Many studies cited concerns about overcorrection for their justification behind not correcting for structure. Interestingly, we did not observe that structure corrections (genetic or spatial) resulted in lower SNP detection rates, suggesting power loss may not be a universal outcome. We concur with previous recommendations that researchers should explore models with and without corrections, particularly in systems with complex demographic histories (Forester, Lasky, et al. 2018; Rellstab et al. 2015). As RDA has proven to be a robust and widely adopted method with relatively low Type I error rates, its continued use as a primary analytical tool is well supported (Capblancq and Forester 2021).

### 4.2 Environmental Drivers of Adaptive Variation Among Taxonomic Groups

We consistently identified that climate variables, particularly measures of extremes and variability, were strongly associated with the detection of putatively adaptive loci. As climate change drives more frequent extreme weather events (Easterling et al. 2000; Thornton et al. 2014), this suggests that many wildlife populations will face intensified selection pressures.

Our review also highlights significant taxonomic biases in the GEA literature; however, these patterns should be interpreted cautiously, as some taxa are represented by multiple analyses and are not fully independent. Ecologically sensitive groups, including herpetofauna and molluscs, are notably underrepresented. For instance, despite their reputation as indicators of ecosystem health (Dietl et al. 2016; Michael et al. 2018; Pruden et al. 2021; Welsh and Droege 2001; Welsh and Ollivier 1998), molluscs and herpetofauna were the focus of only 15 and 23 studies, respectively (Geist 2010). We recommend that future research prioritizes these understudied taxa, as they are crucial for understanding ecosystem health and are likely to be particularly vulnerable to environmental change.

Finally, we confirmed that the drivers of adaptation are highly taxon‐specific. Arthropods showed weak relationships with precipitation and extremes despite the fact that arthropod biomass is strongly associated with precipitation extremes and extreme weather events in many environments (Fischer et al. 2022; Newell et al. 2023; Wise and Lensing 2019). This suggests these conditions are not selective and are instead a constraint, while long term mean and habitat conditions may be more relevant to adaptive responses. Birds displayed overall higher detection rates, and their strong associations with temperature are consistent with well‐documented links between avian physiology and latitudinal temperature gradients (Cossins and Bowler 1987; McPherson et al. 2025). Bony fishes possessed strong relationships with mean environmental regimes, likely reflecting adaptation to persistent conditions (Comte and Olden 2017), while weaker relationships with local variables and extremes may reflect behavioural avoidance to short‐term adverse conditions by vertical or lateral movement (Amat‐Trigo et al. 2023). Mammals showed strong or moderate relationships with most variables, though these patterns may be dominated by migratory ungulates in this review (9 of 29 studies), in which precipitation‐related seasonal resources may shape adaptive responses (Broughton et al. 2008; Soler et al. 2013). The consistently strong relationships between molluscs and all variables are consistent with their limited mobility and direct exposure to both persistent and episodic events (Fortunato 2016). The strong relationships between herpetofauna and extremes may reflect adaptive responses to episodic climatic events, whereas weaker relationships with mean temperature suggest physiological constraints could limit genetic responses to average conditions (Pottier et al. 2025).

### 4.3 Limitations and Recommendations

Our synthesis was constrained by inconsistencies in reporting across the reviewed literature. Key details, such as genomic coverage, candidate loci detected per variable, and methodological rationale (e.g., structure corrections) were often missing, limiting our ability to draw more nuanced conclusions. Additionally, while we categorized the methods used, we did not record details on sample size, design, or documented genetic or spatial structure (although Rellstab et al. 2015 reviews this). We were also unable to test the validity of candidate loci across studies, and future research would benefit greatly from expanding on this limitation by incorporating information on validation approaches in GEA studies. A more quantitative meta‐analysis of outlier statistics, including distributions of scores or *p*‐values across methods and the effects of analytical corrections, could also provide deeper insight into the robustness and transferability of candidate loci. Such work represents an important avenue for future research beyond the scope of this mini review. Despite these limitations, this study provides a brief and focused overview of the current state of GEA research.

## 5 Conclusions

This review synthesizes a decade of rapidly advancing GEA research, revealing key trends in methodology and showcasing the primary drivers of adaptation across animal taxa. The non‐linear relationship between genomic coverage and outlier detection highlights that marker density alone may not guarantee improved inference, reinforcing the importance of thoughtful spatial design across environmental gradients, methodological choices and selection of environmental variables. Across taxa, environmental drivers of adaptive variation were highly variable. The importance of climatic variables and their measurement were frequently associated with candidate loci but varied widely, and in some systems appeared to act more as a demographic constraint than consistent selective pressures. These findings emphasize that GEA outcomes are strongly influenced by organismal biology and adaptive capacity, supporting the use of biologically sound predictor selection and calculation. To move the field forward, standardized reporting is essential for ensuring transparency and enabling future meta‐analyses. By improving reporting standards, using power‐aware and taxon‐focused designs, and aligning predictors with organismal ecology, GEA research can deliver stronger biological insights and provide clear, more reliable guidance for conservation and management in a rapidly changing world.

## Author Contributions

A.K.W. and O.M.R. conceptualized and designed the study. All authors developed the methodology. A.K.W. and O.M.R. conducted the formal analysis and curated the data, and A.K.W. prepared the visualizations. A.K.W. and O.M.R. drafted the manuscript, and all authors contributed to reviewing and editing.

## Funding

The authors have nothing to report.

## Conflicts of Interest

The authors declare no conflicts of interest.

## Supporting information


**File S1:** contains the PRISMA flow diagram.
**File S2:** includes the extracted and cleaned study data, along with methodological summaries and formatted variables used in the analysis.
**File S3:** contains a knitted R Markdown document detailing the analytical workflow and figure generation, which draws on the ‘Cleaning’ and ‘Variables’ sheets from File S2.

## Data Availability

All files required to reproduce these results are provided within the Supporting Information. See Supporting Information for a description of these files.

## References

[mec70328-bib-0001] Amat‐Trigo, F. , D. Andreou , P. K. Gillingham , and J. R. Britton . 2023. “Behavioural Thermoregulation in Cold‐Water Freshwater Fish: Innate Resilience to Climate Warming?” Fish and Fisheries 24, no. 1: 187–195. 10.1111/faf.12720. 37063475 PMC10100141

[mec70328-bib-0002] Balkenhol, N. , R. Y. Dudaniec , K. V. Krutovsky , et al. 2017. “Landscape Genomics: Understanding Relationships Between Environmental Heterogeneity and Genomic Characteristics of Populations.” In Population Genomics, edited by O. P. Rajora , 261–322. Springer International Publishing. 10.1007/13836_2017_2.

[mec70328-bib-0003] Broughton, J. M. , D. A. Byers , R. A. Bryson , W. Eckerle , and D. B. Madsen . 2008. “Did Climatic Seasonality Control Late Quaternary Artiodactyl Densities in Western North America?” Quaternary Science Reviews 27, no. 19–20: 1916–1937. 10.1016/j.quascirev.2008.07.005.

[mec70328-bib-0004] Capblancq, T. , and B. R. Forester . 2021. “Redundancy Analysis: A Swiss Army Knife for Landscape Genomics.” Methods in Ecology and Evolution 12, no. 12: 2298–2309. 10.1111/2041-210X.13722.

[mec70328-bib-0005] Caye, K. , B. Jumentier , J. Lepeule , and O. François . 2019. “LFMM 2: Fast and Accurate Inference of Gene‐Environment Associations in Genome‐Wide Studies.” Molecular Biology and Evolution 36, no. 4: 852–860. 10.1093/molbev/msz008. 30657943 PMC6659841

[mec70328-bib-0006] Comte, L. , and J. D. Olden . 2017. “Evolutionary and Environmental Determinants of Freshwater Fish Thermal Tolerance and Plasticity.” Global Change Biology 23, no. 2: 728–736. 10.1111/gcb.13427. 27406402

[mec70328-bib-0007] Cossins, A. R. , and K. Bowler . 1987. Temperature Biology of Animals. Springer Netherlands. 10.1007/978-94-009-3127-5.

[mec70328-bib-0008] De Mita, S. , A. Thuillet , L. Gay , et al. 2013. “Detecting Selection Along Environmental Gradients: Analysis of Eight Methods and Their Effectiveness for Outbreeding and Selfing Populations.” Molecular Ecology 22, no. 5: 1383–1399. 10.1111/mec.12182. 23294205

[mec70328-bib-0009] Dietl, G. P. , S. R. Durham , J. A. Smith , and A. Tweitmann . 2016. “Mollusk Assemblages as Records of Past and Present Ecological Status.” Frontiers in Marine Science 3: e00169. 10.3389/fmars.2016.00169.

[mec70328-bib-0010] Easterling, D. R. , J. L. Evans , P. Y. Groisman , T. R. Karl , K. E. Kunkel , and P. Ambenje . 2000. “Observed Variability and Trends in Extreme Climate Events: A Brief Review.” Bulletin of the American Meteorological Society 81, no. 3: 417–425. 10.1175/1520-0477(2000)081.

[mec70328-bib-0011] Fischer, C. , R. Gerstmeier , and T. C. Wagner . 2022. “Seasonal and Temporal Patterns of Rainfall Shape Arthropod Community Composition and Multi‐Trophic Interactions in an Arid Environment.” Scientific Reports 12, no. 1: 3742. 10.1038/s41598-022-07716-0. 35260643 PMC8904780

[mec70328-bib-0012] Flanagan, S. P. , B. R. Forester , E. K. Latch , S. N. Aitken , and S. Hoban . 2018. “Guidelines for Planning Genomic Assessment and Monitoring of Locally Adaptive Variation to Inform Species Conservation.” Evolutionary Applications 11, no. 7: 1035–1052. 10.1111/eva.12569. 30026796 PMC6050180

[mec70328-bib-0013] Forester, B. R. , A. S. Cicchino , A. A. Shah , et al. 2025. “Population Genomics Reveals Local Adaptation Related to Temperature Variation in Two Stream Frog Species: Implications for Vulnerability to Climate Warming.” Molecular Ecology 34: e17651. 10.1111/mec.17651. 39825598 PMC12267553

[mec70328-bib-0014] Forester, B. R. , E. L. Landguth , B. K. Hand , and N. Balkenhol . 2018. “Landscape Genomics for Wildlife Research.” In Population Genomics: Wildlife, edited by P. A. Hohenlohe and O. P. Rajora , 145–184. Springer International Publishing. 10.1007/13836_2018_56.

[mec70328-bib-0015] Forester, B. R. , J. R. Lasky , H. H. Wagner , and D. L. Urban . 2018. “Comparing Methods for Detecting Multilocus Adaptation With Multivariate Genotype–Environment Associations.” Molecular Ecology 27, no. 9: 2215–2233. 10.1111/mec.14584. 29633402

[mec70328-bib-0016] Fortunato, H. 2016. “Mollusks: Tools in Environmental and Climate Research*.” American Malacological Bulletin 33, no. 2: 310–324. 10.4003/006.033.0208.

[mec70328-bib-0017] Frichot, E. , S. D. Schoville , G. Bouchard , and O. François . 2013. “Testing for Associations Between Loci and Environmental Gradients Using Latent Factor Mixed Models.” Molecular Biology and Evolution 30, no. 7: 1687–1699. 10.1093/molbev/mst063. 23543094 PMC3684853

[mec70328-bib-0018] Geist, J. 2010. “Strategies for the Conservation of Endangered Freshwater Pearl Mussels ( *Margaritifera margaritifera* L.): A Synthesis of Conservation Genetics and Ecology.” Hydrobiologia 644, no. 1: 69–88. 10.1007/s10750-010-0190-2.

[mec70328-bib-0019] Hoban, S. , J. L. Kelley , K. E. Lotterhos , et al. 2016. “Finding the Genomic Basis of Local Adaptation: Pitfalls, Practical Solutions, and Future Directions.” American Naturalist 188, no. 4: 379–397. 10.1086/688018. PMC5457800 27622873

[mec70328-bib-0020] Lasky, J. R. , E. B. Josephs , and G. P. Morris . 2023. “Genotype–Environment Associations to Reveal the Molecular Basis of Environmental Adaptation.” Plant Cell 35, no. 1: 125–138. 10.1093/plcell/koac267. 36005926 PMC9806588

[mec70328-bib-0021] Lotterhos, K. E. , and M. C. Whitlock . 2014. “Evaluation of Demographic History and Neutral Parameterization on the Performance of * f * _ st _ Outlier Tests.” Molecular Ecology 23, no. 9: 2178–2192. 10.1111/mec.12725. 24655127 PMC4228763

[mec70328-bib-0022] McPherson, R. A. , K. E. Alger , and E. Hofmeister . 2025. “Climate‐Related Drivers of Migratory Bird Health in the South‐Central usa .” Biological Reviews 100, no. 3: 1272–1293. 10.1111/brv.70000. 39912288 PMC12120389

[mec70328-bib-0023] Michael, D. R. , M. Crane , D. Florance , and D. B. Lindenmayer . 2018. “Revegetation, Restoration and Reptiles in Rural Landscapes: Insights From Long‐Term Monitoring Programmes in the Temperate Eucalypt Woodlands of South‐Eastern Australia.” Ecological Management & Restoration 19, no. 1: 32–38. 10.1111/emr.12294.

[mec70328-bib-0024] Newell, F. L. , I. J. Ausprey , and S. K. Robinson . 2023. “Wet and Dry Extremes Reduce Arthropod Biomass Independently of Leaf Phenology in the Wet Tropics.” Global Change Biology 29, no. 2: 308–323. 10.1111/gcb.16379. 36102197 PMC10087840

[mec70328-bib-0025] Page, M. J. , J. E. McKenzie , P. M. Bossuyt , et al. 2021. “The PRISMA 2020 Statement: An Updated Guideline for Reporting Systematic Reviews.” BMJ 372: n71. 10.1136/bmj.n71. 33782057 PMC8005924

[mec70328-bib-0026] Pottier, P. , M. R. Kearney , N. C. Wu , et al. 2025. “Vulnerability of Amphibians to Global Warming.” Nature 639, no. 8056: 954–961. 10.1038/s41586-025-08665-0. 40044855 PMC11946914

[mec70328-bib-0027] Pruden, M. J. , G. P. Dietl , J. C. Handley , and J. A. Smith . 2021. “Using Molluscs to Assess Ecological Quality Status of Soft‐Bottom Habitats Along the Atlantic Coastline of the United States.” Ecological Indicators 129: 107910. 10.1016/j.ecolind.2021.107910.

[mec70328-bib-0028] R Core Team . 2024. R: A Language and Environment for Statistical Computing (Version 4.4.2) [Computer Software]. R Foundation for Statistical Computing.

[mec70328-bib-0029] Razgour, O. , J. B. Taggart , S. Manel , et al. 2018. “An Integrated Framework to Identify Wildlife Populations Under Threat From Climate Change.” Molecular Ecology Resources 18, no. 1: 18–31. 10.1111/1755-0998.12694. 28649779 PMC6849758

[mec70328-bib-0030] Rellstab, C. , F. Gugerli , A. J. Eckert , A. M. Hancock , and R. Holderegger . 2015. “A Practical Guide to Environmental Association Analysis in Landscape Genomics.” Molecular Ecology 24, no. 17: 4348–4370. 10.1111/mec.13322. 26184487

[mec70328-bib-0031] RStudio Team . 2024. Rstudio: Integrated Development for R. (Version 2024.12.0) [Computer Software]. RStudio.

[mec70328-bib-0032] Soler, R. M. , G. Martínez Pastur , M. V. Lencinas , and L. Borrelli . 2013. “Seasonal Diet of *Lama guanicoe* (Camelidae: Artiodactyla) in a Heterogeneous Landscape of South Patagonia.” Bosque 34, no. 2: 3–4. 10.4067/S0717-92002013000200002.

[mec70328-bib-0033] Thornton, P. K. , P. J. Ericksen , M. Herrero , and A. J. Challinor . 2014. “Climate Variability and Vulnerability to Climate Change: A Review.” Global Change Biology 20, no. 11: 3313–3328. 10.1111/gcb.12581. 24668802 PMC4258067

[mec70328-bib-0034] Welsh, H. H. , and S. Droege . 2001. “A Case for Using Plethodontid Salamanders for Monitoring Biodiversity and Ecosystem Integrity of North American Forests.” Conservation Biology 15, no. 3: 558–569. 10.1046/j.1523-1739.2001.015003558.x.

[mec70328-bib-0035] Welsh, H. H. , and L. M. Ollivier . 1998. “Stream Amphibians as Indicators of Ecosystem Stress: A Case Study From California's Redwoods.” Ecological Applications 8, no. 4: 1118–1132. 10.1890/1051-0761(1998)008[1118:SAAIOE]2.0.CO;2.

[mec70328-bib-0036] Wickham, H. , M. Averick , J. Bryan , et al. 2019. “Welcome to the Tidyverse.” Journal of Open Source Software 4, no. 43: 1686. 10.21105/joss.01686.

[mec70328-bib-0037] Wise, D. H. , and J. R. Lensing . 2019. “Impacts of Rainfall Extremes Predicted by Climate‐Change Models on Major Trophic Groups in the Leaf Litter Arthropod Community.” Journal of Animal Ecology 88, no. 10: 1486–1497. 10.1111/1365-2656.13046. 31211860

