# Supplementary material for: Climate Extremes, Genomic Coverage, and Taxonomy Shape the Detection of Adaptation: A Systematic Review of GEA Studies for the Kingdom Animalia
Source: Mol Ecol. 2026 Mar 25;35(6):e70328. doi: 10.1111/mec.70328 (PMC13017192; doi:10.1111/mec.70328)
Supplement: Supplementary file 1 — File S1: contains the PRISMA flow diagram. File S2: includes the extracted and cleaned study data, along with methodological summaries and formatted variables used in the analysis. File S3: contains a knitted R Markdown document detailing the analytical workflow and figure generation, which draws on the ‘Cleaning’ and ‘Variables’ sheets from File S2. [file MEC-35-e70328-s001.zip › SI_File3.html]

Manuscript\_Figures


Code 

- Download Rmd

# Manuscript\_Figures

#### 2026-01-27

Library and Data

```
library(tidyverse)
```

```
## Warning: package 'ggplot2' was built under R version 4.5.2
```

```
## ── Attaching core tidyverse packages ──────────────────────── tidyverse 2.0.0 ──
## ✔ dplyr     1.1.4     ✔ readr     2.1.5
## ✔ forcats   1.0.0     ✔ stringr   1.5.1
## ✔ ggplot2   4.0.1     ✔ tibble    3.3.0
## ✔ lubridate 1.9.4     ✔ tidyr     1.3.1
## ✔ purrr     1.0.4     
## ── Conflicts ────────────────────────────────────────── tidyverse_conflicts() ──
## ✖ dplyr::filter() masks stats::filter()
## ✖ dplyr::lag()    masks stats::lag()
## ℹ Use the conflicted package (<http://conflicted.r-lib.org/>) to force all conflicts to become errors
```

```
library(scales)
```

```
## 
## Attaching package: 'scales'
## 
## The following object is masked from 'package:purrr':
## 
##     discard
## 
## The following object is masked from 'package:readr':
## 
##     col_factor
```

```
library(patchwork)
library(readxl)
library(forcats)
library(MASS)
```

```
## 
## Attaching package: 'MASS'
## 
## The following object is masked from 'package:patchwork':
## 
##     area
## 
## The following object is masked from 'package:dplyr':
## 
##     select
```

```
library(knitr)
library(kableExtra)
```

```
## Warning: package 'kableExtra' was built under R version 4.5.2
```

```
## 
## Attaching package: 'kableExtra'
## 
## The following object is masked from 'package:dplyr':
## 
##     group_rows
```

```
# by sheet name
df <- read_excel("SI_File2_Review.xlsx", sheet = "Cleaning")
```

# 1 Misc. summary Results

- Number of unique studies and methods
- Number of studies using PCA for variable reduction
- Mean number of outliers detected by 1 method

  - Mean number of outliers shared across >1 method

```
# How many unique studies?
length(unique(df$Study_ID))
```

```
## [1] 194
```

```
# How many analyses?
length(df$Study_ID)
```

```
## [1] 343
```

```
# avg analyses per study:
length(df$Study_ID)/length(unique(df$Study_ID))
```

```
## [1] 1.768041
```

```
# mean markers used per study
df$No_SNPs <- as.numeric(df$No_SNPs)
```

```
## Warning: NAs introduced by coercion
```

```
mean(df$No_SNPs, na.rm = TRUE)
```

```
## [1] 1072683
```

```
sd(df$No_SNPs, na.rm = TRUE)
```

```
## [1] 4431838
```

```
# mean loci detected per study and shared
df$outliers_detected <- as.numeric(df$outliers_detected)
```

```
## Warning: NAs introduced by coercion
```

```
df$no_outliers_shared <- as.numeric(df$no_outliers_shared)
```

```
## Warning: NAs introduced by coercion
```

```
mean(df$outliers_detected, na.rm = TRUE)
```

```
## [1] 1977.316
```

```
sd(df$outliers_detected, na.rm = TRUE)
```

```
## [1] 6067.129
```

```
mean(df$no_outliers_shared, na.rm = TRUE)
```

```
## [1] 656.5056
```

```
sd(df$no_outliers_shared, na.rm = TRUE)
```

```
## [1] 2559.183
```

```
# mean loci shared across multiple studies

# average genomic coverage
df$genome_coverage <- df$No_SNPs/(df$Genome_size*1e9)
mean(df$genome_coverage, na.rm = TRUE)
```

```
## [1] 0.0008187228
```

```
sd(df$genome_coverage, na.rm = TRUE)
```

```
## [1] 0.002781085
```

```
# marker type
df |> 
  group_by(marker_origin) |>
  summarise(
  n = n()
)
```

```
## # A tibble: 8 × 2
##   marker_origin                                                     n
##   <chr>                                                         <int>
## 1 DArT-based reduced-representation sequencing                      8
## 2 Hybridization-based capture sequencing (targeted loci)            6
## 3 Restriction-enzyme–based reduced-representation sequencing      223
## 4 SNP arrays / chip-based genotyping                                9
## 5 Targeted SNP or amplicon panels                                  10
## 6 Transcriptomic / metagenomic sequencing (non-genomic markers)     7
## 7 Whole-genome shotgun–based approaches                            78
## 8 unclear                                                           2
```

```
# PCA for dimensionality reduction
length(which(df$PC_axes_as_predictors=="Y"))/343
```

```
## [1] 0.180758
```

# 2 Study Corrections

- Summarizing corrections for structure

```
# Correction summary:

df <- df |> 
  mutate(
    Year = as.factor(Year),
    #outliers_detected = na_if(outliers_detected, "NR"),
    outliers_detected = as.numeric(outliers_detected),
    No_SNPs = as.numeric(No_SNPs)
  )

df_corr <- df %>%
  mutate(
    pop_corr = `account for gen. pop. structure?`,
    geo_corr = `account for geographic structure?`,
    combo = paste0(geo_corr, pop_corr)  # geo first, then pop: YY, YN, NY, NN
  )

summary_combo <- df_corr %>%
  count(combo, name = "n") %>%
  complete(combo = c("YY","YN","NY","NN"), fill = list(n = 0)) %>%
  mutate(
    prop = n / sum(n),
    pct  = 100 * prop
  ) %>%
  arrange(match(combo, c("YY","YN","NY","NN")))

summary_combo
```

```
## # A tibble: 4 × 4
##   combo     n   prop   pct
##   <chr> <int>  <dbl> <dbl>
## 1 YY       31 0.0904  9.04
## 2 YN       46 0.134  13.4 
## 3 NY      162 0.472  47.2 
## 4 NN      104 0.303  30.3
```

Now which methods specifically account for structure:

```
dat_filt <- df %>%
  dplyr::select(1, 13, 16, 15, 18, 21, 24) %>%
  mutate(
    outliers_detected = as.numeric(outliers_detected),
    No_SNPs = as.numeric(No_SNPs),
    SNP_det_rate = outliers_detected / No_SNPs,
    geo = `account for geographic structure?`,
    pop = `account for gen. pop. structure?`,
    combo = paste0(geo, pop)
  )
```

Summarize functions

```
summ_stats <- function(data) {
  data %>%
    summarise(
      mean = mean(SNP_det_rate, na.rm = TRUE),
      sd   = sd(SNP_det_rate, na.rm = TRUE),
      n    = sum(!is.na(SNP_det_rate)),
      .groups = "drop"
    )
}
```

Make summary tables of corrections

```
by_method <- dat_filt %>%
  group_by(combo, GEA_method) %>%
  summ_stats() %>%
  ungroup()

overall <- dat_filt %>%
  group_by(combo) %>%
  summ_stats() %>%
  mutate(GEA_method = "Overall") %>%
  dplyr::select(combo, GEA_method, dplyr::everything())

summary_tbl <- bind_rows(by_method, overall) %>%
  mutate(
    combo = factor(combo, levels = c("YY","YN","NY","NN"))
  ) %>%
  arrange(combo, desc(GEA_method == "Overall"), GEA_method)
```

Table plotting

```
summary_tbl %>%
  kable(digits = 4, booktabs = TRUE,
        col.names = c("Structure combo (geo/pop)", "GEA method", "Mean", "SD", "n")) %>%
  kable_styling(full_width = FALSE)
```

| Structure combo (geo/pop) | GEA method | Mean | SD | n |
| --- | --- | --- | --- | --- |
| YY | Overall | 0.0403 | 0.0694 | 30 |
| YY | BayPass | 0.0107 | 0.0059 | 4 |
| YY | BayeScEnv | 0.0021 | NA | 1 |
| YY | Bayenv / Bayenv2 | 0.0304 | NA | 1 |
| YY | GEMMA | NaN | NA | 0 |
| YY | LFMM | 0.0678 | 0.1049 | 9 |
| YY | RDA | 0.0086 | 0.0145 | 3 |
| YY | SAMβADA | 0.1365 | NA | 1 |
| YY | Samβada | 0.0055 | NA | 1 |
| YY | gINLAnd | 0.0637 | NA | 1 |
| YY | gradient forest | 0.0042 | NA | 1 |
| YY | linear mixed model | 0.0007 | NA | 1 |
| YY | pRDA | 0.0412 | 0.0609 | 7 |
| YN | Overall | 0.0224 | 0.0230 | 42 |
| YN | BayeScEnv | NaN | NA | 0 |
| YN | LFMM | 0.0056 | 0.0025 | 3 |
| YN | Pearson correlations | 0.0015 | NA | 1 |
| YN | RDA | 0.0188 | 0.0145 | 14 |
| YN | Samβada | 0.1161 | NA | 1 |
| YN | gINLAnd | 0.0182 | 0.0154 | 4 |
| YN | pRDA | 0.0247 | 0.0208 | 19 |
| NY | Overall | 0.0346 | 0.0601 | 140 |
| NY | ANGSD-asso latent model | 0.0003 | 0.0003 | 2 |
| NY | BayPass | 0.0569 | 0.1113 | 18 |
| NY | BayeScEnv | 0.0148 | 0.0154 | 4 |
| NY | Bayenv / Bayenv2 | 0.0390 | 0.0653 | 13 |
| NY | Gradient forest | 0.0035 | NA | 1 |
| NY | LFMM | 0.0325 | 0.0510 | 71 |
| NY | Mixed effect logistic regression model | 0.0099 | NA | 1 |
| NY | RDA | 0.0274 | 0.0390 | 6 |
| NY | SAMβADA | 0.0012 | NA | 1 |
| NY | Samβada | 0.0231 | 0.0298 | 2 |
| NY | gINLAnd | 0.0086 | NA | 1 |
| NY | gradient forest | NaN | NA | 0 |
| NY | pRDA | 0.0353 | 0.0408 | 20 |
| NN | Overall | 0.0203 | 0.0281 | 88 |
| NN | BayeScEnv | 0.0008 | 0.0012 | 2 |
| NN | Bayenv / Bayenv2 | 0.0140 | 0.0173 | 2 |
| NN | Gradient forest | 0.0518 | 0.0579 | 4 |
| NN | LFMM | 0.0312 | 0.0660 | 5 |
| NN | Moran Spectral Outlier Detection | 0.0315 | NA | 1 |
| NN | Moran spectral randomization | 0.0182 | NA | 1 |
| NN | RDA | 0.0173 | 0.0207 | 64 |
| NN | SAMβADA | 0.0402 | NA | 1 |
| NN | Samβada | 0.0000 | NA | 1 |
| NN | linear mixed-effect model | 0.0317 | NA | 1 |
| NN | multiple regression on distance matrices | 0.0755 | NA | 1 |
| NN | pRDA | 0.0187 | 0.0298 | 5 |
| NN | random forest | NaN | NA | 0 |

Boxplots

```
ggplot(dat_filt %>% drop_na(SNP_det_rate),
       aes(x = combo, y = SNP_det_rate)) +
  geom_boxplot(outlier.alpha = 0.3) +
  labs(
    x = "Structure correction (Geo / Pop)",
    y = "SNP detection rate"
  ) +
  theme_classic()
```

Regardless of corrections, were some methods more effective at
detecting outliers?

```
dat_filt %>%
  filter(!is.na(SNP_det_rate), !is.na(GEA_method)) %>%
  mutate(
    GEA_method = reorder(
      GEA_method,
      SNP_det_rate,
      FUN = median,
      na.rm = TRUE
    )
  ) %>%
  ggplot(aes(x = GEA_method, y = SNP_det_rate)) +
  geom_boxplot(outlier.alpha = 0.25) +
  coord_flip() +
  labs(
    x = "GEA method",
    y = "SNP detection rate"
  ) +
  theme_classic()
```

```
# method grouping
dat_filt %>%
  filter(!is.na(SNP_det_rate), !is.na(Method_category)) %>%
  mutate(
    Method_category = reorder(
      Method_category,
      SNP_det_rate,
      FUN = median,
      na.rm = TRUE
    )
  ) %>%
  ggplot(aes(x = Method_category, y = SNP_det_rate)) +
  geom_boxplot(outlier.alpha = 0.25) +
  coord_flip() +
  labs(
    x = "Method category",
    y = "SNP detection rate"
  ) +
  theme_classic()
```

# 3 Figure 1

## 3.1 a)

```
# Histogram
hist_dat <- df |> 
  mutate(Year = as.factor(Year)) |>
  distinct(Study_ID, .keep_all = TRUE)

a <- 
  ggplot(hist_dat, aes(x = Year)) + geom_histogram(stat = "count") + 
  labs(y = "No. RDA studies", title = "a") + 
  annotate("text",
           x = "2025",
           y = 18,
           label = "*",
           size = 5) +
  theme_minimal(10) +
  theme(axis.text.x = element_text(angle = 45, hjust = 1, vjust = 1, size = 8),
        axis.text.y = element_text(size = 8))
```

```
## Warning in geom_histogram(stat = "count"): Ignoring unknown parameters:
## `binwidth` and `bins`
```

```
a
```

## 3.2 b)

```
# FILTER OUTLIER STUDIES
# FILTER TO GENOME-REPRESENTATIVE SOURCES
df_b <- df |>
  dplyr::filter(!is.na(outliers_detected), 
                !is.na(No_SNPs), 
                !Study_ID %in% c("29", "53"), 
                !marker_origin == "Transcriptomic / metagenomic sequencing (non-genomic markers)",
                !marker_origin == "Hybridization-based capture sequencing (targeted loci)",
                !marker_origin == "Targeted SNP or amplicon panels") |>
  dplyr::mutate(raw_size = Genome_size * 1e9, sampled_prop = No_SNPs / raw_size)

m_nb_lin <- glm.nb(outliers_detected ~ sampled_prop, data=df_b)
m_nb_quad <- glm.nb(outliers_detected ~ sampled_prop + I(sampled_prop^2), data=df_b)
anova(m_nb_lin, m_nb_quad, test="Chisq"); AIC(m_nb_lin, m_nb_quad)
```

```
## Likelihood ratio tests of Negative Binomial Models
## 
## Response: outliers_detected
##                              Model     theta Resid. df    2 x log-lik.   Test
## 1                     sampled_prop 0.3741281       278       -4292.492       
## 2 sampled_prop + I(sampled_prop^2) 0.4081041       277       -4257.884 1 vs 2
##      df LR stat.      Pr(Chi)
## 1                            
## 2     1 34.60779 4.032898e-09
```

```
##           df      AIC
## m_nb_lin   3 4298.492
## m_nb_quad  4 4265.884
```

```
co <- coef(m_nb_quad); b1 <- co["sampled_prop"]; b2 <- co["I(sampled_prop^2)"]
x_peak_nb <- -b1/(2*b2)
x_peak_nb <- if(is.finite(x_peak_nb) && b2 < 0 && x_peak_nb >= min(df_b$sampled_prop) && x_peak_nb <= max(df_b$sampled_prop)) x_peak_nb else NA_real_
peak_df <- data.frame(sampled_prop=x_peak_nb, label=paste0("Peak: ", scales::percent(x_peak_nb, accuracy=0.01)))

mean_gc <- mean(df_b$sampled_prop, na.rm = TRUE)  # 0.04% average genomic coverage (as a proportion)
mean_df <- data.frame(sampled_prop=mean_gc, label=paste0("Mean: ", scales::percent(mean_gc, accuracy=0.01)))

y_top <- max(df_b$outliers_detected, na.rm=TRUE)

b <- ggplot(df_b, aes(x=sampled_prop, y=outliers_detected)) +
  geom_point(size=0.7, alpha=0.5) +
  stat_smooth(method="glm.nb", formula=y~x+I(x^2), se=TRUE, linewidth=0.8, alpha=0.25) +
  geom_vline(data=mean_df, aes(xintercept=sampled_prop), linetype="dashed", color="red") +
  geom_vline(data=peak_df, aes(xintercept=sampled_prop), linetype="dotted", linewidth=0.9, color="darkblue", na.rm=TRUE) +
  theme_minimal(10) +
  scale_x_continuous(labels=scales::percent_format(accuracy=0.01)) +
  labs(x="Proportion of genome sampled", y="No. outliers detected", title="b")

b
```

```
df_b |>
  summarise(
    mean_prop_genome = mean(sampled_prop, na.rm = TRUE),
    sd_prop_genome   = sd(sampled_prop, na.rm = TRUE),
    n                = sum(!is.na(sampled_prop))
  )
```

```
## # A tibble: 1 × 3
##   mean_prop_genome sd_prop_genome     n
##              <dbl>          <dbl> <int>
## 1         0.000482        0.00139   280
```

```
length(which(df_b$genome_coverage > 0.0045))
```

```
## [1] 11
```

```
length(which(df_b$genome_coverage > 0.0015))/length(df_b$genome_coverage)
```

```
## [1] 0.09642857
```

```
length(which(df_b$genome_coverage > 0.0034))/length(df_b$genome_coverage)
```

```
## [1] 0.04642857
```

Repeat b for shared outliers, see if genomic coverage relationship
varies…

```
df_b <- df |>
  dplyr::filter(!is.na(no_outliers_shared), !is.na(No_SNPs), !Study_ID %in% c("29", "53")) |>
  dplyr::mutate(raw_size = Genome_size * 1e9, sampled_prop = No_SNPs / raw_size)

m_nb_lin <- glm.nb(no_outliers_shared ~ sampled_prop, data=df_b)
m_nb_quad <- glm.nb(no_outliers_shared ~ sampled_prop + I(sampled_prop^2), data=df_b)
anova(m_nb_lin, m_nb_quad, test="Chisq"); AIC(m_nb_lin, m_nb_quad)
```

```
## Likelihood ratio tests of Negative Binomial Models
## 
## Response: no_outliers_shared
##                              Model     theta Resid. df    2 x log-lik.   Test
## 1                     sampled_prop 0.3338890        85       -989.3541       
## 2 sampled_prop + I(sampled_prop^2) 0.3595995        84       -980.9949 1 vs 2
##      df LR stat.     Pr(Chi)
## 1                           
## 2     1 8.359169 0.003837461
```

```
##           df      AIC
## m_nb_lin   3 995.3541
## m_nb_quad  4 988.9949
```

```
co <- coef(m_nb_quad); b1 <- co["sampled_prop"]; b2 <- co["I(sampled_prop^2)"]
x_peak_nb <- -b1/(2*b2)
x_peak_nb <- if(is.finite(x_peak_nb) && b2 < 0 && x_peak_nb >= min(df_b$sampled_prop) && x_peak_nb <= max(df_b$sampled_prop)) x_peak_nb else NA_real_
peak_df <- data.frame(sampled_prop=x_peak_nb, label=paste0("Peak: ", scales::percent(x_peak_nb, accuracy=0.01)))

mean_gc <- mean(df_b$sampled_prop, na.rm = TRUE)  # 0.04% average genomic coverage (as a proportion)
mean_df <- data.frame(sampled_prop=mean_gc, label=paste0("Mean: ", scales::percent(mean_gc, accuracy=0.01)))

y_top <- max(df_b$outliers_detected, na.rm=TRUE)

b_shared <- ggplot(df_b, aes(x=sampled_prop, y=no_outliers_shared)) +
  geom_point(size=0.7, alpha=0.5) +
  stat_smooth(method="glm.nb", formula=y~x+I(x^2), se=TRUE, linewidth=0.8, alpha=0.25) +
  geom_vline(data=mean_df, aes(xintercept=sampled_prop), linetype="dashed", color="red") +
  geom_vline(data=peak_df, aes(xintercept=sampled_prop), linetype="dotted", linewidth=0.9, color="darkblue", na.rm=TRUE) +
  theme_minimal(10) +
  scale_x_continuous(labels=scales::percent_format(accuracy=0.01)) +
  labs(x="Proportion of genome sampled", y="No. outliers detected", title="b")

b_shared
```

```
df_b |>
  summarise(
    mean_prop_genome = mean(sampled_prop, na.rm = TRUE),
    sd_prop_genome   = sd(sampled_prop, na.rm = TRUE),
    n                = sum(!is.na(sampled_prop))
  )
```

```
## # A tibble: 1 × 3
##   mean_prop_genome sd_prop_genome     n
##              <dbl>          <dbl> <int>
## 1         0.000689        0.00204    87
```

## 3.3 c)

```
# Methods bar plot

methods_clean <- df %>%
  count(GEA_method, name = "Count") %>%
  mutate(GEA_method = ifelse(Count < 5, "Other*", GEA_method)) %>%
  group_by(GEA_method) %>%
  summarise(Count = sum(Count), .groups = "drop") %>%
  arrange(desc(Count)) %>%
  mutate(
    Method = fct_reorder(GEA_method, Count, .desc = TRUE),
    Method = fct_relevel(Method, "Other*", after = Inf)
  )
methods_clean <- methods_clean %>%
  mutate(Method = recode(Method,
                         "Bayenv / Bayenv2" = "Bayenv(2)"))
c <- 
  ggplot(methods_clean, aes(x = Method, y = Count)) +
  geom_bar(stat = "identity") +
  theme_minimal(10) +
  labs(x = "Method", y = "Count", title = "c") +
  scale_x_discrete(labels = function(x) str_wrap(x, width = 15)) +
  theme(
    legend.position = "none",
    axis.text.x = element_text(angle = 45, hjust = 0.8, size = 8),
    axis.text.y = element_text(size = 8)
  )

c
```

## 3.4 d)

```
# d. Broad taxa. group

orders_summed <- df |>
  count(Broader, name = "TotalCount") |>
  mutate(Broader = fct_reorder(Broader, TotalCount, .desc = TRUE))

# Now plot with the reordered factor
d <-
  ggplot(orders_summed, aes(x = Broader, y = TotalCount)) +
  geom_bar(stat = "identity") +  # "identity" because we already summed Count
  theme_minimal(base_size = 10) +
  labs(x = "Broad taxonomic group", y = "Count", title = "d") +
  scale_x_discrete(labels = function(x) str_wrap(x, width = 10)) +
  theme(legend.position = "none",
        axis.text.x = element_text(angle = 45, hjust = 0.8, size = 8),
        axis.text.y = element_text(size = 8)) 

d
```

```
ggsave("Fig1a-d.pdf",
       plot = ((a + b) + plot_layout(widths = c(1, 1.5))) /
         ((c + d) + plot_layout(widths = c(1, 1.5))),
       device = cairo_pdf,   # best text rendering
       width = 170, height = 150, units = "mm")
```

# 4 Figure 2

## 4.1 Prepare files and functions

```
file <- read_excel("SI_File2_Review.xlsx", sheet = "Variables")
eps <- 1e-6  # small constant so log10 works if SNP_det_rate can be 0

# remove groups with less than 10 observations
# Read once + compute log10 SNP detection rate
base_dat <- file |>
  mutate(
    Broader = stringr::str_squish(Broader),
    SNP_det_rate = parse_number(as.character(SNP_det_rate)),
    SNP_log = log10(SNP_det_rate + eps)
  ) |>
  filter(!is.na(SNP_det_rate),
         Broader != "Annelids",
         Broader != "Cnidarians",
         Broader != "Tunicates",
         Broader != "Echinoderms",
         Broader != "Jawless Fishes")
```

## 4.2 Helper functions and plotting code

```
# Manual legend order (and locked color mapping)
broader_levels <- c(
  "Arthropods", "Birds", "Bony Fishes", 
  "Mammals", "Mollusks", "Reptiles & Amphibians"
)

broader_pal <- scales::hue_pal()(length(broader_levels))
names(broader_pal) <- broader_levels

# Helper: IQR filter + n>5 within (x_var, Broader), returning a clean plot-ready df
prep_plot_dat <- function(dat, x_var) {
  dat |>
    mutate(
      x_raw = str_trim(tolower(.data[[x_var]])),
      x_plot = na_if(x_raw, "")
    ) |>
    filter(!is.na(x_plot),
           !x_plot %in% c("na", "n/a", "n.a.", "nan")) |>
    group_by(x_plot, Broader) |>
    mutate(
      q1  = quantile(SNP_log, 0.25, na.rm = TRUE),
      q3  = quantile(SNP_log, 0.75, na.rm = TRUE),
      iqr = q3 - q1,
      lo  = q1 - 1.5 * iqr,
      hi  = q3 + 1.5 * iqr
    ) |>
    filter(between(SNP_log, lo, hi)) |>
    filter(n() > 5) |>
    ungroup()
}

# Helper: overall mean + 95% CI (on log scale) by x_plot
overall_ci <- function(dat) {
  dat |>
    group_by(x_plot) |>
    summarise(
      mean = mean(SNP_log, na.rm = TRUE),
      sd   = sd(SNP_log, na.rm = TRUE),
      n    = n(),
      se   = sd / sqrt(n),
      lwr  = mean - 1.96 * se,
      upr  = mean + 1.96 * se,
      .groups = "drop"
    )
}

# Helper: build the ggplot (box + jitter + overall mean/CI)
make_plot <- function(dat, overall, title_letter, xlab_wrap = 10) {
  ggplot(dat, aes(x = x_plot, y = SNP_log, color = Broader)) +
    geom_boxplot(
      position = position_dodge(width = 0.75),
      width = 0.6,
      linewidth = 0.3,
      outlier.shape = NA
    ) +
    geom_jitter(
      position = position_jitterdodge(
        jitter.width = 0.15,
        dodge.width = 0.75
      ),
      size = 0.3,
      alpha = 0.7
    ) +
    geom_errorbar(
      data = overall,
      aes(x = x_plot, ymin = lwr, ymax = upr),
      width = 0.2,
      inherit.aes = FALSE,
      color = "black"
    ) +
    geom_point(
      data = overall,
      aes(x = x_plot, y = mean),
      inherit.aes = FALSE,
      color = "black",
      size = 1.5
    ) +
    scale_x_discrete(labels = function(x) str_wrap(x, width = xlab_wrap)) +
    scale_color_manual(values = broader_pal, breaks = broader_levels, drop = FALSE) +
    theme_minimal(10) +
    labs(title = title_letter, x = "", y = "") +
    theme(
      axis.text.x = element_text(size = 8),
      axis.text.y = element_text(size = 8),
      legend.position = "none"
    )
}
```

## 4.3 a, b, and c:

```
# Plot A: variable.grouping
plot_a_dat <- prep_plot_dat(base_dat, "variable.grouping")
plot_a_dat <- plot_a_dat |> mutate(Broader = factor(Broader, levels = broader_levels))

# Your desired order for A (edit as needed)
var_order_a <- c(
  "temp. mean", "temp. ext.", "temp. var.",
  "precip. annual", "precip. qu.", "precip. ext.", "precip. var.",
  "prod. mean", "prod. ext.",
  "water cond. mean", "hab. cond.",
  "topo./hydro.", "spatial"
)

plot_a_dat <- plot_a_dat |>
  mutate(x_plot = factor(x_plot, levels = var_order_a))

# FILTER TOPO AND SPACE so other categories can be more easily visualized. 
# These categories have low numbers of observations and only 1 taxonomic group
plot_a_dat <- plot_a_dat |>
  filter(!x_plot %in% c("topo./hydro.", "spatial"))

overall_a <- overall_ci(plot_a_dat)
a <- make_plot(plot_a_dat, overall_a, "a") + guides(color = "none")

a
```

```
# Plot B: variable_type
plot_b_dat <- prep_plot_dat(base_dat, "variable_type")
plot_b_dat <- plot_b_dat |> mutate(Broader = factor(Broader, levels = broader_levels))

# Your desired order for B (edit as needed)
var_order_b <- c("temp.", "precip.", "water cond.", "prod.", "site")

plot_b_dat <- plot_b_dat |>
  mutate(x_plot = factor(x_plot, levels = var_order_b))

overall_b <- overall_ci(plot_b_dat)
b <- make_plot(plot_b_dat, overall_b, "b") +
  theme(legend.position = "right") + labs( y = "log10(SNP detection rate)")

b
```

```
# Plot C: measurement_summary_type

plot_c_dat <- prep_plot_dat(base_dat, "measurement_summary_type")
plot_c_dat <- plot_c_dat |> mutate(Broader = factor(Broader, levels = broader_levels))

# Fill in later:
var_order_c <- c(
  "mean", "extreme", "variation", "raw"
)
plot_c_dat <- plot_c_dat |>
   mutate(x_plot = factor(x_plot, levels = var_order_c))

overall_c <- overall_ci(plot_c_dat)
c <- make_plot(plot_c_dat, overall_c, "c") + guides(color = "none") +
  labs(x = "Variable category")

c
```

Save

```
combined <- (a / b / c) +
  plot_layout(guides = "collect", heights = c(1, 1, 1)) &
  theme(
    legend.position = "bottom",
    legend.title = element_blank(),
    plot.margin = margin(0, 0, 0, 0)
  )

combined
```

```
## Warning: annotation$theme is not a valid theme.
## Please use `theme()` to construct themes.
```

```
ggsave("Fig2a-c.pdf",
       plot = combined,
       device = cairo_pdf,   # best text rendering
       width = 174, height = 230, units = "mm")
```

```
## Warning: annotation$theme is not a valid theme.
## Please use `theme()` to construct themes.
```

LS0tDQp0aXRsZTogIk1hbnVzY3JpcHRfRmlndXJlcyINCmRhdGU6ICIyMDI2LTAxLTI3Ig0Kb3V0cHV0OiANCiAgaHRtbF9kb2N1bWVudDoNCiAgICB0b2M6IHRydWUNCiAgICB0b2NfZmxvYXQ6IHRydWUNCiAgICBudW1iZXJfc2VjdGlvbnM6IHRydWUNCiAgICB0aGVtZTogZmxhdGx5DQogICAgY29kZV9kb3dubG9hZDogdHJ1ZQ0KZWRpdG9yX29wdGlvbnM6IA0KICBtYXJrZG93bjogDQogICAgd3JhcDogNzINCi0tLQ0KDQpMaWJyYXJ5IGFuZCBEYXRhDQoNCmBgYHtyIHNldHVwfQ0KbGlicmFyeSh0aWR5dmVyc2UpDQpsaWJyYXJ5KHNjYWxlcykNCmxpYnJhcnkocGF0Y2h3b3JrKQ0KbGlicmFyeShyZWFkeGwpDQpsaWJyYXJ5KGZvcmNhdHMpDQpsaWJyYXJ5KE1BU1MpDQpsaWJyYXJ5KGtuaXRyKQ0KbGlicmFyeShrYWJsZUV4dHJhKQ0KDQojIGJ5IHNoZWV0IG5hbWUNCmRmIDwtIHJlYWRfZXhjZWwoIlNJX0ZpbGUyX1Jldmlldy54bHN4Iiwgc2hlZXQgPSAiQ2xlYW5pbmciKQ0KYGBgDQoNCiMgTWlzYy4gc3VtbWFyeSBSZXN1bHRzDQoNCi0gICBOdW1iZXIgb2YgdW5pcXVlIHN0dWRpZXMgYW5kIG1ldGhvZHMNCg0KLSAgIE51bWJlciBvZiBzdHVkaWVzIHVzaW5nIFBDQSBmb3IgdmFyaWFibGUgcmVkdWN0aW9uDQoNCi0gICBNZWFuIG51bWJlciBvZiBvdXRsaWVycyBkZXRlY3RlZCBieSAxIG1ldGhvZA0KDQogICAgLSAgIE1lYW4gbnVtYmVyIG9mIG91dGxpZXJzIHNoYXJlZCBhY3Jvc3MgXD4xIG1ldGhvZA0KDQpgYGB7cn0NCiMgSG93IG1hbnkgdW5pcXVlIHN0dWRpZXM/DQpsZW5ndGgodW5pcXVlKGRmJFN0dWR5X0lEKSkNCg0KIyBIb3cgbWFueSBhbmFseXNlcz8NCmxlbmd0aChkZiRTdHVkeV9JRCkNCg0KIyBhdmcgYW5hbHlzZXMgcGVyIHN0dWR5Og0KbGVuZ3RoKGRmJFN0dWR5X0lEKS9sZW5ndGgodW5pcXVlKGRmJFN0dWR5X0lEKSkNCg0KIyBtZWFuIG1hcmtlcnMgdXNlZCBwZXIgc3R1ZHkNCmRmJE5vX1NOUHMgPC0gYXMubnVtZXJpYyhkZiROb19TTlBzKQ0KbWVhbihkZiROb19TTlBzLCBuYS5ybSA9IFRSVUUpDQpzZChkZiROb19TTlBzLCBuYS5ybSA9IFRSVUUpDQoNCiMgbWVhbiBsb2NpIGRldGVjdGVkIHBlciBzdHVkeSBhbmQgc2hhcmVkDQpkZiRvdXRsaWVyc19kZXRlY3RlZCA8LSBhcy5udW1lcmljKGRmJG91dGxpZXJzX2RldGVjdGVkKQ0KZGYkbm9fb3V0bGllcnNfc2hhcmVkIDwtIGFzLm51bWVyaWMoZGYkbm9fb3V0bGllcnNfc2hhcmVkKQ0KDQptZWFuKGRmJG91dGxpZXJzX2RldGVjdGVkLCBuYS5ybSA9IFRSVUUpDQpzZChkZiRvdXRsaWVyc19kZXRlY3RlZCwgbmEucm0gPSBUUlVFKQ0KDQptZWFuKGRmJG5vX291dGxpZXJzX3NoYXJlZCwgbmEucm0gPSBUUlVFKQ0Kc2QoZGYkbm9fb3V0bGllcnNfc2hhcmVkLCBuYS5ybSA9IFRSVUUpDQoNCiMgbWVhbiBsb2NpIHNoYXJlZCBhY3Jvc3MgbXVsdGlwbGUgc3R1ZGllcw0KDQojIGF2ZXJhZ2UgZ2Vub21pYyBjb3ZlcmFnZQ0KZGYkZ2Vub21lX2NvdmVyYWdlIDwtIGRmJE5vX1NOUHMvKGRmJEdlbm9tZV9zaXplKjFlOSkNCm1lYW4oZGYkZ2Vub21lX2NvdmVyYWdlLCBuYS5ybSA9IFRSVUUpDQpzZChkZiRnZW5vbWVfY292ZXJhZ2UsIG5hLnJtID0gVFJVRSkNCg0KIyBtYXJrZXIgdHlwZQ0KZGYgfD4gDQogIGdyb3VwX2J5KG1hcmtlcl9vcmlnaW4pIHw+DQogIHN1bW1hcmlzZSgNCiAgbiA9IG4oKQ0KKQ0KDQojIFBDQSBmb3IgZGltZW5zaW9uYWxpdHkgcmVkdWN0aW9uDQpsZW5ndGgod2hpY2goZGYkUENfYXhlc19hc19wcmVkaWN0b3JzPT0iWSIpKS8zNDMNCg0KYGBgDQoNCiMgU3R1ZHkgQ29ycmVjdGlvbnMNCg0KLSAgIFN1bW1hcml6aW5nIGNvcnJlY3Rpb25zIGZvciBzdHJ1Y3R1cmUNCg0KYGBge3J9DQojIENvcnJlY3Rpb24gc3VtbWFyeToNCg0KZGYgPC0gZGYgfD4gDQogIG11dGF0ZSgNCiAgICBZZWFyID0gYXMuZmFjdG9yKFllYXIpLA0KICAgICNvdXRsaWVyc19kZXRlY3RlZCA9IG5hX2lmKG91dGxpZXJzX2RldGVjdGVkLCAiTlIiKSwNCiAgICBvdXRsaWVyc19kZXRlY3RlZCA9IGFzLm51bWVyaWMob3V0bGllcnNfZGV0ZWN0ZWQpLA0KICAgIE5vX1NOUHMgPSBhcy5udW1lcmljKE5vX1NOUHMpDQogICkNCg0KZGZfY29yciA8LSBkZiAlPiUNCiAgbXV0YXRlKA0KICAgIHBvcF9jb3JyID0gYGFjY291bnQgZm9yIGdlbi4gcG9wLiBzdHJ1Y3R1cmU/YCwNCiAgICBnZW9fY29yciA9IGBhY2NvdW50IGZvciBnZW9ncmFwaGljIHN0cnVjdHVyZT9gLA0KICAgIGNvbWJvID0gcGFzdGUwKGdlb19jb3JyLCBwb3BfY29ycikgICMgZ2VvIGZpcnN0LCB0aGVuIHBvcDogWVksIFlOLCBOWSwgTk4NCiAgKQ0KDQpzdW1tYXJ5X2NvbWJvIDwtIGRmX2NvcnIgJT4lDQogIGNvdW50KGNvbWJvLCBuYW1lID0gIm4iKSAlPiUNCiAgY29tcGxldGUoY29tYm8gPSBjKCJZWSIsIllOIiwiTlkiLCJOTiIpLCBmaWxsID0gbGlzdChuID0gMCkpICU+JQ0KICBtdXRhdGUoDQogICAgcHJvcCA9IG4gLyBzdW0obiksDQogICAgcGN0ICA9IDEwMCAqIHByb3ANCiAgKSAlPiUNCiAgYXJyYW5nZShtYXRjaChjb21ibywgYygiWVkiLCJZTiIsIk5ZIiwiTk4iKSkpDQoNCnN1bW1hcnlfY29tYm8NCg0KYGBgDQoNCk5vdyB3aGljaCBtZXRob2RzIHNwZWNpZmljYWxseSBhY2NvdW50IGZvciBzdHJ1Y3R1cmU6DQoNCmBgYHtyfQ0KZGF0X2ZpbHQgPC0gZGYgJT4lDQogIGRwbHlyOjpzZWxlY3QoMSwgMTMsIDE2LCAxNSwgMTgsIDIxLCAyNCkgJT4lDQogIG11dGF0ZSgNCiAgICBvdXRsaWVyc19kZXRlY3RlZCA9IGFzLm51bWVyaWMob3V0bGllcnNfZGV0ZWN0ZWQpLA0KICAgIE5vX1NOUHMgPSBhcy5udW1lcmljKE5vX1NOUHMpLA0KICAgIFNOUF9kZXRfcmF0ZSA9IG91dGxpZXJzX2RldGVjdGVkIC8gTm9fU05QcywNCiAgICBnZW8gPSBgYWNjb3VudCBmb3IgZ2VvZ3JhcGhpYyBzdHJ1Y3R1cmU/YCwNCiAgICBwb3AgPSBgYWNjb3VudCBmb3IgZ2VuLiBwb3AuIHN0cnVjdHVyZT9gLA0KICAgIGNvbWJvID0gcGFzdGUwKGdlbywgcG9wKQ0KICApDQpgYGANCg0KU3VtbWFyaXplIGZ1bmN0aW9ucw0KDQpgYGB7cn0NCnN1bW1fc3RhdHMgPC0gZnVuY3Rpb24oZGF0YSkgew0KICBkYXRhICU+JQ0KICAgIHN1bW1hcmlzZSgNCiAgICAgIG1lYW4gPSBtZWFuKFNOUF9kZXRfcmF0ZSwgbmEucm0gPSBUUlVFKSwNCiAgICAgIHNkICAgPSBzZChTTlBfZGV0X3JhdGUsIG5hLnJtID0gVFJVRSksDQogICAgICBuICAgID0gc3VtKCFpcy5uYShTTlBfZGV0X3JhdGUpKSwNCiAgICAgIC5ncm91cHMgPSAiZHJvcCINCiAgICApDQp9DQpgYGANCg0KTWFrZSBzdW1tYXJ5IHRhYmxlcyBvZiBjb3JyZWN0aW9ucw0KDQpgYGB7cn0NCmJ5X21ldGhvZCA8LSBkYXRfZmlsdCAlPiUNCiAgZ3JvdXBfYnkoY29tYm8sIEdFQV9tZXRob2QpICU+JQ0KICBzdW1tX3N0YXRzKCkgJT4lDQogIHVuZ3JvdXAoKQ0KDQpvdmVyYWxsIDwtIGRhdF9maWx0ICU+JQ0KICBncm91cF9ieShjb21ibykgJT4lDQogIHN1bW1fc3RhdHMoKSAlPiUNCiAgbXV0YXRlKEdFQV9tZXRob2QgPSAiT3ZlcmFsbCIpICU+JQ0KICBkcGx5cjo6c2VsZWN0KGNvbWJvLCBHRUFfbWV0aG9kLCBkcGx5cjo6ZXZlcnl0aGluZygpKQ0KDQpzdW1tYXJ5X3RibCA8LSBiaW5kX3Jvd3MoYnlfbWV0aG9kLCBvdmVyYWxsKSAlPiUNCiAgbXV0YXRlKA0KICAgIGNvbWJvID0gZmFjdG9yKGNvbWJvLCBsZXZlbHMgPSBjKCJZWSIsIllOIiwiTlkiLCJOTiIpKQ0KICApICU+JQ0KICBhcnJhbmdlKGNvbWJvLCBkZXNjKEdFQV9tZXRob2QgPT0gIk92ZXJhbGwiKSwgR0VBX21ldGhvZCkNCmBgYA0KDQpUYWJsZSBwbG90dGluZw0KDQpgYGB7cn0NCnN1bW1hcnlfdGJsICU+JQ0KICBrYWJsZShkaWdpdHMgPSA0LCBib29rdGFicyA9IFRSVUUsDQogICAgICAgIGNvbC5uYW1lcyA9IGMoIlN0cnVjdHVyZSBjb21ibyAoZ2VvL3BvcCkiLCAiR0VBIG1ldGhvZCIsICJNZWFuIiwgIlNEIiwgIm4iKSkgJT4lDQogIGthYmxlX3N0eWxpbmcoZnVsbF93aWR0aCA9IEZBTFNFKQ0KYGBgDQoNCkJveHBsb3RzDQoNCmBgYHtyfQ0KZ2dwbG90KGRhdF9maWx0ICU+JSBkcm9wX25hKFNOUF9kZXRfcmF0ZSksDQogICAgICAgYWVzKHggPSBjb21ibywgeSA9IFNOUF9kZXRfcmF0ZSkpICsNCiAgZ2VvbV9ib3hwbG90KG91dGxpZXIuYWxwaGEgPSAwLjMpICsNCiAgbGFicygNCiAgICB4ID0gIlN0cnVjdHVyZSBjb3JyZWN0aW9uIChHZW8gLyBQb3ApIiwNCiAgICB5ID0gIlNOUCBkZXRlY3Rpb24gcmF0ZSINCiAgKSArDQogIHRoZW1lX2NsYXNzaWMoKQ0KYGBgDQoNClJlZ2FyZGxlc3Mgb2YgY29ycmVjdGlvbnMsIHdlcmUgc29tZSBtZXRob2RzIG1vcmUgZWZmZWN0aXZlIGF0IGRldGVjdGluZw0Kb3V0bGllcnM/DQoNCmBgYHtyfQ0KZGF0X2ZpbHQgJT4lDQogIGZpbHRlcighaXMubmEoU05QX2RldF9yYXRlKSwgIWlzLm5hKEdFQV9tZXRob2QpKSAlPiUNCiAgbXV0YXRlKA0KICAgIEdFQV9tZXRob2QgPSByZW9yZGVyKA0KICAgICAgR0VBX21ldGhvZCwNCiAgICAgIFNOUF9kZXRfcmF0ZSwNCiAgICAgIEZVTiA9IG1lZGlhbiwNCiAgICAgIG5hLnJtID0gVFJVRQ0KICAgICkNCiAgKSAlPiUNCiAgZ2dwbG90KGFlcyh4ID0gR0VBX21ldGhvZCwgeSA9IFNOUF9kZXRfcmF0ZSkpICsNCiAgZ2VvbV9ib3hwbG90KG91dGxpZXIuYWxwaGEgPSAwLjI1KSArDQogIGNvb3JkX2ZsaXAoKSArDQogIGxhYnMoDQogICAgeCA9ICJHRUEgbWV0aG9kIiwNCiAgICB5ID0gIlNOUCBkZXRlY3Rpb24gcmF0ZSINCiAgKSArDQogIHRoZW1lX2NsYXNzaWMoKQ0KDQojIG1ldGhvZCBncm91cGluZw0KZGF0X2ZpbHQgJT4lDQogIGZpbHRlcighaXMubmEoU05QX2RldF9yYXRlKSwgIWlzLm5hKE1ldGhvZF9jYXRlZ29yeSkpICU+JQ0KICBtdXRhdGUoDQogICAgTWV0aG9kX2NhdGVnb3J5ID0gcmVvcmRlcigNCiAgICAgIE1ldGhvZF9jYXRlZ29yeSwNCiAgICAgIFNOUF9kZXRfcmF0ZSwNCiAgICAgIEZVTiA9IG1lZGlhbiwNCiAgICAgIG5hLnJtID0gVFJVRQ0KICAgICkNCiAgKSAlPiUNCiAgZ2dwbG90KGFlcyh4ID0gTWV0aG9kX2NhdGVnb3J5LCB5ID0gU05QX2RldF9yYXRlKSkgKw0KICBnZW9tX2JveHBsb3Qob3V0bGllci5hbHBoYSA9IDAuMjUpICsNCiAgY29vcmRfZmxpcCgpICsNCiAgbGFicygNCiAgICB4ID0gIk1ldGhvZCBjYXRlZ29yeSIsDQogICAgeSA9ICJTTlAgZGV0ZWN0aW9uIHJhdGUiDQogICkgKw0KICB0aGVtZV9jbGFzc2ljKCkNCmBgYA0KDQojIEZpZ3VyZSAxDQoNCiMjIGEpDQoNCmBgYHtyfQ0KIyBIaXN0b2dyYW0NCmhpc3RfZGF0IDwtIGRmIHw+IA0KICBtdXRhdGUoWWVhciA9IGFzLmZhY3RvcihZZWFyKSkgfD4NCiAgZGlzdGluY3QoU3R1ZHlfSUQsIC5rZWVwX2FsbCA9IFRSVUUpDQoNCmEgPC0gDQogIGdncGxvdChoaXN0X2RhdCwgYWVzKHggPSBZZWFyKSkgKyBnZW9tX2hpc3RvZ3JhbShzdGF0ID0gImNvdW50IikgKyANCiAgbGFicyh5ID0gIk5vLiBSREEgc3R1ZGllcyIsIHRpdGxlID0gImEiKSArIA0KICBhbm5vdGF0ZSgidGV4dCIsDQogICAgICAgICAgIHggPSAiMjAyNSIsDQogICAgICAgICAgIHkgPSAxOCwNCiAgICAgICAgICAgbGFiZWwgPSAiKiIsDQogICAgICAgICAgIHNpemUgPSA1KSArDQogIHRoZW1lX21pbmltYWwoMTApICsNCiAgdGhlbWUoYXhpcy50ZXh0LnggPSBlbGVtZW50X3RleHQoYW5nbGUgPSA0NSwgaGp1c3QgPSAxLCB2anVzdCA9IDEsIHNpemUgPSA4KSwNCiAgICAgICAgYXhpcy50ZXh0LnkgPSBlbGVtZW50X3RleHQoc2l6ZSA9IDgpKQ0KDQphDQpgYGANCg0KIyMgYikNCg0KYGBge3J9DQojIEZJTFRFUiBPVVRMSUVSIFNUVURJRVMNCiMgRklMVEVSIFRPIEdFTk9NRS1SRVBSRVNFTlRBVElWRSBTT1VSQ0VTDQpkZl9iIDwtIGRmIHw+DQogIGRwbHlyOjpmaWx0ZXIoIWlzLm5hKG91dGxpZXJzX2RldGVjdGVkKSwgDQogICAgICAgICAgICAgICAgIWlzLm5hKE5vX1NOUHMpLCANCiAgICAgICAgICAgICAgICAhU3R1ZHlfSUQgJWluJSBjKCIyOSIsICI1MyIpLCANCiAgICAgICAgICAgICAgICAhbWFya2VyX29yaWdpbiA9PSAiVHJhbnNjcmlwdG9taWMgLyBtZXRhZ2Vub21pYyBzZXF1ZW5jaW5nIChub24tZ2Vub21pYyBtYXJrZXJzKSIsDQogICAgICAgICAgICAgICAgIW1hcmtlcl9vcmlnaW4gPT0gIkh5YnJpZGl6YXRpb24tYmFzZWQgY2FwdHVyZSBzZXF1ZW5jaW5nICh0YXJnZXRlZCBsb2NpKSIsDQogICAgICAgICAgICAgICAgIW1hcmtlcl9vcmlnaW4gPT0gIlRhcmdldGVkIFNOUCBvciBhbXBsaWNvbiBwYW5lbHMiKSB8Pg0KICBkcGx5cjo6bXV0YXRlKHJhd19zaXplID0gR2Vub21lX3NpemUgKiAxZTksIHNhbXBsZWRfcHJvcCA9IE5vX1NOUHMgLyByYXdfc2l6ZSkNCg0KbV9uYl9saW4gPC0gZ2xtLm5iKG91dGxpZXJzX2RldGVjdGVkIH4gc2FtcGxlZF9wcm9wLCBkYXRhPWRmX2IpDQptX25iX3F1YWQgPC0gZ2xtLm5iKG91dGxpZXJzX2RldGVjdGVkIH4gc2FtcGxlZF9wcm9wICsgSShzYW1wbGVkX3Byb3BeMiksIGRhdGE9ZGZfYikNCmFub3ZhKG1fbmJfbGluLCBtX25iX3F1YWQsIHRlc3Q9IkNoaXNxIik7IEFJQyhtX25iX2xpbiwgbV9uYl9xdWFkKQ0KDQpjbyA8LSBjb2VmKG1fbmJfcXVhZCk7IGIxIDwtIGNvWyJzYW1wbGVkX3Byb3AiXTsgYjIgPC0gY29bIkkoc2FtcGxlZF9wcm9wXjIpIl0NCnhfcGVha19uYiA8LSAtYjEvKDIqYjIpDQp4X3BlYWtfbmIgPC0gaWYoaXMuZmluaXRlKHhfcGVha19uYikgJiYgYjIgPCAwICYmIHhfcGVha19uYiA+PSBtaW4oZGZfYiRzYW1wbGVkX3Byb3ApICYmIHhfcGVha19uYiA8PSBtYXgoZGZfYiRzYW1wbGVkX3Byb3ApKSB4X3BlYWtfbmIgZWxzZSBOQV9yZWFsXw0KcGVha19kZiA8LSBkYXRhLmZyYW1lKHNhbXBsZWRfcHJvcD14X3BlYWtfbmIsIGxhYmVsPXBhc3RlMCgiUGVhazogIiwgc2NhbGVzOjpwZXJjZW50KHhfcGVha19uYiwgYWNjdXJhY3k9MC4wMSkpKQ0KDQptZWFuX2djIDwtIG1lYW4oZGZfYiRzYW1wbGVkX3Byb3AsIG5hLnJtID0gVFJVRSkgICMgMC4wNCUgYXZlcmFnZSBnZW5vbWljIGNvdmVyYWdlIChhcyBhIHByb3BvcnRpb24pDQptZWFuX2RmIDwtIGRhdGEuZnJhbWUoc2FtcGxlZF9wcm9wPW1lYW5fZ2MsIGxhYmVsPXBhc3RlMCgiTWVhbjogIiwgc2NhbGVzOjpwZXJjZW50KG1lYW5fZ2MsIGFjY3VyYWN5PTAuMDEpKSkNCg0KeV90b3AgPC0gbWF4KGRmX2Ikb3V0bGllcnNfZGV0ZWN0ZWQsIG5hLnJtPVRSVUUpDQoNCmIgPC0gZ2dwbG90KGRmX2IsIGFlcyh4PXNhbXBsZWRfcHJvcCwgeT1vdXRsaWVyc19kZXRlY3RlZCkpICsNCiAgZ2VvbV9wb2ludChzaXplPTAuNywgYWxwaGE9MC41KSArDQogIHN0YXRfc21vb3RoKG1ldGhvZD0iZ2xtLm5iIiwgZm9ybXVsYT15fngrSSh4XjIpLCBzZT1UUlVFLCBsaW5ld2lkdGg9MC44LCBhbHBoYT0wLjI1KSArDQogIGdlb21fdmxpbmUoZGF0YT1tZWFuX2RmLCBhZXMoeGludGVyY2VwdD1zYW1wbGVkX3Byb3ApLCBsaW5ldHlwZT0iZGFzaGVkIiwgY29sb3I9InJlZCIpICsNCiAgZ2VvbV92bGluZShkYXRhPXBlYWtfZGYsIGFlcyh4aW50ZXJjZXB0PXNhbXBsZWRfcHJvcCksIGxpbmV0eXBlPSJkb3R0ZWQiLCBsaW5ld2lkdGg9MC45LCBjb2xvcj0iZGFya2JsdWUiLCBuYS5ybT1UUlVFKSArDQogIHRoZW1lX21pbmltYWwoMTApICsNCiAgc2NhbGVfeF9jb250aW51b3VzKGxhYmVscz1zY2FsZXM6OnBlcmNlbnRfZm9ybWF0KGFjY3VyYWN5PTAuMDEpKSArDQogIGxhYnMoeD0iUHJvcG9ydGlvbiBvZiBnZW5vbWUgc2FtcGxlZCIsIHk9Ik5vLiBvdXRsaWVycyBkZXRlY3RlZCIsIHRpdGxlPSJiIikNCg0KYg0KDQpkZl9iIHw+DQogIHN1bW1hcmlzZSgNCiAgICBtZWFuX3Byb3BfZ2Vub21lID0gbWVhbihzYW1wbGVkX3Byb3AsIG5hLnJtID0gVFJVRSksDQogICAgc2RfcHJvcF9nZW5vbWUgICA9IHNkKHNhbXBsZWRfcHJvcCwgbmEucm0gPSBUUlVFKSwNCiAgICBuICAgICAgICAgICAgICAgID0gc3VtKCFpcy5uYShzYW1wbGVkX3Byb3ApKQ0KICApDQoNCmxlbmd0aCh3aGljaChkZl9iJGdlbm9tZV9jb3ZlcmFnZSA+IDAuMDA0NSkpDQoNCmxlbmd0aCh3aGljaChkZl9iJGdlbm9tZV9jb3ZlcmFnZSA+IDAuMDAxNSkpL2xlbmd0aChkZl9iJGdlbm9tZV9jb3ZlcmFnZSkNCg0KbGVuZ3RoKHdoaWNoKGRmX2IkZ2Vub21lX2NvdmVyYWdlID4gMC4wMDM0KSkvbGVuZ3RoKGRmX2IkZ2Vub21lX2NvdmVyYWdlKQ0KDQpgYGANCg0KDQpSZXBlYXQgYiBmb3Igc2hhcmVkIG91dGxpZXJzLCBzZWUgaWYgZ2Vub21pYyBjb3ZlcmFnZSByZWxhdGlvbnNoaXAgdmFyaWVzLi4uDQpgYGB7cn0NCmRmX2IgPC0gZGYgfD4NCiAgZHBseXI6OmZpbHRlcighaXMubmEobm9fb3V0bGllcnNfc2hhcmVkKSwgIWlzLm5hKE5vX1NOUHMpLCAhU3R1ZHlfSUQgJWluJSBjKCIyOSIsICI1MyIpKSB8Pg0KICBkcGx5cjo6bXV0YXRlKHJhd19zaXplID0gR2Vub21lX3NpemUgKiAxZTksIHNhbXBsZWRfcHJvcCA9IE5vX1NOUHMgLyByYXdfc2l6ZSkNCg0KbV9uYl9saW4gPC0gZ2xtLm5iKG5vX291dGxpZXJzX3NoYXJlZCB+IHNhbXBsZWRfcHJvcCwgZGF0YT1kZl9iKQ0KbV9uYl9xdWFkIDwtIGdsbS5uYihub19vdXRsaWVyc19zaGFyZWQgfiBzYW1wbGVkX3Byb3AgKyBJKHNhbXBsZWRfcHJvcF4yKSwgZGF0YT1kZl9iKQ0KYW5vdmEobV9uYl9saW4sIG1fbmJfcXVhZCwgdGVzdD0iQ2hpc3EiKTsgQUlDKG1fbmJfbGluLCBtX25iX3F1YWQpDQoNCmNvIDwtIGNvZWYobV9uYl9xdWFkKTsgYjEgPC0gY29bInNhbXBsZWRfcHJvcCJdOyBiMiA8LSBjb1siSShzYW1wbGVkX3Byb3BeMikiXQ0KeF9wZWFrX25iIDwtIC1iMS8oMipiMikNCnhfcGVha19uYiA8LSBpZihpcy5maW5pdGUoeF9wZWFrX25iKSAmJiBiMiA8IDAgJiYgeF9wZWFrX25iID49IG1pbihkZl9iJHNhbXBsZWRfcHJvcCkgJiYgeF9wZWFrX25iIDw9IG1heChkZl9iJHNhbXBsZWRfcHJvcCkpIHhfcGVha19uYiBlbHNlIE5BX3JlYWxfDQpwZWFrX2RmIDwtIGRhdGEuZnJhbWUoc2FtcGxlZF9wcm9wPXhfcGVha19uYiwgbGFiZWw9cGFzdGUwKCJQZWFrOiAiLCBzY2FsZXM6OnBlcmNlbnQoeF9wZWFrX25iLCBhY2N1cmFjeT0wLjAxKSkpDQoNCm1lYW5fZ2MgPC0gbWVhbihkZl9iJHNhbXBsZWRfcHJvcCwgbmEucm0gPSBUUlVFKSAgIyAwLjA0JSBhdmVyYWdlIGdlbm9taWMgY292ZXJhZ2UgKGFzIGEgcHJvcG9ydGlvbikNCm1lYW5fZGYgPC0gZGF0YS5mcmFtZShzYW1wbGVkX3Byb3A9bWVhbl9nYywgbGFiZWw9cGFzdGUwKCJNZWFuOiAiLCBzY2FsZXM6OnBlcmNlbnQobWVhbl9nYywgYWNjdXJhY3k9MC4wMSkpKQ0KDQp5X3RvcCA8LSBtYXgoZGZfYiRvdXRsaWVyc19kZXRlY3RlZCwgbmEucm09VFJVRSkNCg0KYl9zaGFyZWQgPC0gZ2dwbG90KGRmX2IsIGFlcyh4PXNhbXBsZWRfcHJvcCwgeT1ub19vdXRsaWVyc19zaGFyZWQpKSArDQogIGdlb21fcG9pbnQoc2l6ZT0wLjcsIGFscGhhPTAuNSkgKw0KICBzdGF0X3Ntb290aChtZXRob2Q9ImdsbS5uYiIsIGZvcm11bGE9eX54K0koeF4yKSwgc2U9VFJVRSwgbGluZXdpZHRoPTAuOCwgYWxwaGE9MC4yNSkgKw0KICBnZW9tX3ZsaW5lKGRhdGE9bWVhbl9kZiwgYWVzKHhpbnRlcmNlcHQ9c2FtcGxlZF9wcm9wKSwgbGluZXR5cGU9ImRhc2hlZCIsIGNvbG9yPSJyZWQiKSArDQogIGdlb21fdmxpbmUoZGF0YT1wZWFrX2RmLCBhZXMoeGludGVyY2VwdD1zYW1wbGVkX3Byb3ApLCBsaW5ldHlwZT0iZG90dGVkIiwgbGluZXdpZHRoPTAuOSwgY29sb3I9ImRhcmtibHVlIiwgbmEucm09VFJVRSkgKw0KICB0aGVtZV9taW5pbWFsKDEwKSArDQogIHNjYWxlX3hfY29udGludW91cyhsYWJlbHM9c2NhbGVzOjpwZXJjZW50X2Zvcm1hdChhY2N1cmFjeT0wLjAxKSkgKw0KICBsYWJzKHg9IlByb3BvcnRpb24gb2YgZ2Vub21lIHNhbXBsZWQiLCB5PSJOby4gb3V0bGllcnMgZGV0ZWN0ZWQiLCB0aXRsZT0iYiIpDQoNCmJfc2hhcmVkDQoNCmRmX2IgfD4NCiAgc3VtbWFyaXNlKA0KICAgIG1lYW5fcHJvcF9nZW5vbWUgPSBtZWFuKHNhbXBsZWRfcHJvcCwgbmEucm0gPSBUUlVFKSwNCiAgICBzZF9wcm9wX2dlbm9tZSAgID0gc2Qoc2FtcGxlZF9wcm9wLCBuYS5ybSA9IFRSVUUpLA0KICAgIG4gICAgICAgICAgICAgICAgPSBzdW0oIWlzLm5hKHNhbXBsZWRfcHJvcCkpDQogICkNCmBgYA0KDQoNCiMjIGMpDQoNCmBgYHtyfQ0KIyBNZXRob2RzIGJhciBwbG90DQoNCm1ldGhvZHNfY2xlYW4gPC0gZGYgJT4lDQogIGNvdW50KEdFQV9tZXRob2QsIG5hbWUgPSAiQ291bnQiKSAlPiUNCiAgbXV0YXRlKEdFQV9tZXRob2QgPSBpZmVsc2UoQ291bnQgPCA1LCAiT3RoZXIqIiwgR0VBX21ldGhvZCkpICU+JQ0KICBncm91cF9ieShHRUFfbWV0aG9kKSAlPiUNCiAgc3VtbWFyaXNlKENvdW50ID0gc3VtKENvdW50KSwgLmdyb3VwcyA9ICJkcm9wIikgJT4lDQogIGFycmFuZ2UoZGVzYyhDb3VudCkpICU+JQ0KICBtdXRhdGUoDQogICAgTWV0aG9kID0gZmN0X3Jlb3JkZXIoR0VBX21ldGhvZCwgQ291bnQsIC5kZXNjID0gVFJVRSksDQogICAgTWV0aG9kID0gZmN0X3JlbGV2ZWwoTWV0aG9kLCAiT3RoZXIqIiwgYWZ0ZXIgPSBJbmYpDQogICkNCm1ldGhvZHNfY2xlYW4gPC0gbWV0aG9kc19jbGVhbiAlPiUNCiAgbXV0YXRlKE1ldGhvZCA9IHJlY29kZShNZXRob2QsDQogICAgICAgICAgICAgICAgICAgICAgICAgIkJheWVudiAvIEJheWVudjIiID0gIkJheWVudigyKSIpKQ0KYyA8LSANCiAgZ2dwbG90KG1ldGhvZHNfY2xlYW4sIGFlcyh4ID0gTWV0aG9kLCB5ID0gQ291bnQpKSArDQogIGdlb21fYmFyKHN0YXQgPSAiaWRlbnRpdHkiKSArDQogIHRoZW1lX21pbmltYWwoMTApICsNCiAgbGFicyh4ID0gIk1ldGhvZCIsIHkgPSAiQ291bnQiLCB0aXRsZSA9ICJjIikgKw0KICBzY2FsZV94X2Rpc2NyZXRlKGxhYmVscyA9IGZ1bmN0aW9uKHgpIHN0cl93cmFwKHgsIHdpZHRoID0gMTUpKSArDQogIHRoZW1lKA0KICAgIGxlZ2VuZC5wb3NpdGlvbiA9ICJub25lIiwNCiAgICBheGlzLnRleHQueCA9IGVsZW1lbnRfdGV4dChhbmdsZSA9IDQ1LCBoanVzdCA9IDAuOCwgc2l6ZSA9IDgpLA0KICAgIGF4aXMudGV4dC55ID0gZWxlbWVudF90ZXh0KHNpemUgPSA4KQ0KICApDQoNCmMNCmBgYA0KDQojIyBkKQ0KDQpgYGB7cn0NCiMgZC4gQnJvYWQgdGF4YS4gZ3JvdXANCg0Kb3JkZXJzX3N1bW1lZCA8LSBkZiB8Pg0KICBjb3VudChCcm9hZGVyLCBuYW1lID0gIlRvdGFsQ291bnQiKSB8Pg0KICBtdXRhdGUoQnJvYWRlciA9IGZjdF9yZW9yZGVyKEJyb2FkZXIsIFRvdGFsQ291bnQsIC5kZXNjID0gVFJVRSkpDQoNCiMgTm93IHBsb3Qgd2l0aCB0aGUgcmVvcmRlcmVkIGZhY3Rvcg0KZCA8LQ0KICBnZ3Bsb3Qob3JkZXJzX3N1bW1lZCwgYWVzKHggPSBCcm9hZGVyLCB5ID0gVG90YWxDb3VudCkpICsNCiAgZ2VvbV9iYXIoc3RhdCA9ICJpZGVudGl0eSIpICsgICMgImlkZW50aXR5IiBiZWNhdXNlIHdlIGFscmVhZHkgc3VtbWVkIENvdW50DQogIHRoZW1lX21pbmltYWwoYmFzZV9zaXplID0gMTApICsNCiAgbGFicyh4ID0gIkJyb2FkIHRheG9ub21pYyBncm91cCIsIHkgPSAiQ291bnQiLCB0aXRsZSA9ICJkIikgKw0KICBzY2FsZV94X2Rpc2NyZXRlKGxhYmVscyA9IGZ1bmN0aW9uKHgpIHN0cl93cmFwKHgsIHdpZHRoID0gMTApKSArDQogIHRoZW1lKGxlZ2VuZC5wb3NpdGlvbiA9ICJub25lIiwNCiAgICAgICAgYXhpcy50ZXh0LnggPSBlbGVtZW50X3RleHQoYW5nbGUgPSA0NSwgaGp1c3QgPSAwLjgsIHNpemUgPSA4KSwNCiAgICAgICAgYXhpcy50ZXh0LnkgPSBlbGVtZW50X3RleHQoc2l6ZSA9IDgpKSANCg0KZA0KYGBgDQoNCmBgYHtyfQ0KZ2dzYXZlKCJGaWcxYS1kLnBkZiIsDQogICAgICAgcGxvdCA9ICgoYSArIGIpICsgcGxvdF9sYXlvdXQod2lkdGhzID0gYygxLCAxLjUpKSkgLw0KICAgICAgICAgKChjICsgZCkgKyBwbG90X2xheW91dCh3aWR0aHMgPSBjKDEsIDEuNSkpKSwNCiAgICAgICBkZXZpY2UgPSBjYWlyb19wZGYsICAgIyBiZXN0IHRleHQgcmVuZGVyaW5nDQogICAgICAgd2lkdGggPSAxNzAsIGhlaWdodCA9IDE1MCwgdW5pdHMgPSAibW0iKQ0KYGBgDQoNCiMgRmlndXJlIDINCg0KIyMgUHJlcGFyZSBmaWxlcyBhbmQgZnVuY3Rpb25zDQoNCmBgYHtyfQ0KZmlsZSA8LSByZWFkX2V4Y2VsKCJTSV9GaWxlMl9SZXZpZXcueGxzeCIsIHNoZWV0ID0gIlZhcmlhYmxlcyIpDQplcHMgPC0gMWUtNiAgIyBzbWFsbCBjb25zdGFudCBzbyBsb2cxMCB3b3JrcyBpZiBTTlBfZGV0X3JhdGUgY2FuIGJlIDANCg0KIyByZW1vdmUgZ3JvdXBzIHdpdGggbGVzcyB0aGFuIDEwIG9ic2VydmF0aW9ucw0KIyBSZWFkIG9uY2UgKyBjb21wdXRlIGxvZzEwIFNOUCBkZXRlY3Rpb24gcmF0ZQ0KYmFzZV9kYXQgPC0gZmlsZSB8Pg0KICBtdXRhdGUoDQogICAgQnJvYWRlciA9IHN0cmluZ3I6OnN0cl9zcXVpc2goQnJvYWRlciksDQogICAgU05QX2RldF9yYXRlID0gcGFyc2VfbnVtYmVyKGFzLmNoYXJhY3RlcihTTlBfZGV0X3JhdGUpKSwNCiAgICBTTlBfbG9nID0gbG9nMTAoU05QX2RldF9yYXRlICsgZXBzKQ0KICApIHw+DQogIGZpbHRlcighaXMubmEoU05QX2RldF9yYXRlKSwNCiAgICAgICAgIEJyb2FkZXIgIT0gIkFubmVsaWRzIiwNCiAgICAgICAgIEJyb2FkZXIgIT0gIkNuaWRhcmlhbnMiLA0KICAgICAgICAgQnJvYWRlciAhPSAiVHVuaWNhdGVzIiwNCiAgICAgICAgIEJyb2FkZXIgIT0gIkVjaGlub2Rlcm1zIiwNCiAgICAgICAgIEJyb2FkZXIgIT0gIkphd2xlc3MgRmlzaGVzIikNCmBgYA0KDQojIyBIZWxwZXIgZnVuY3Rpb25zIGFuZCBwbG90dGluZyBjb2RlDQoNCmBgYHtyfQ0KIyBNYW51YWwgbGVnZW5kIG9yZGVyIChhbmQgbG9ja2VkIGNvbG9yIG1hcHBpbmcpDQpicm9hZGVyX2xldmVscyA8LSBjKA0KICAiQXJ0aHJvcG9kcyIsICJCaXJkcyIsICJCb255IEZpc2hlcyIsIA0KICAiTWFtbWFscyIsICJNb2xsdXNrcyIsICJSZXB0aWxlcyAmIEFtcGhpYmlhbnMiDQopDQoNCmJyb2FkZXJfcGFsIDwtIHNjYWxlczo6aHVlX3BhbCgpKGxlbmd0aChicm9hZGVyX2xldmVscykpDQpuYW1lcyhicm9hZGVyX3BhbCkgPC0gYnJvYWRlcl9sZXZlbHMNCg0KIyBIZWxwZXI6IElRUiBmaWx0ZXIgKyBuPjUgd2l0aGluICh4X3ZhciwgQnJvYWRlciksIHJldHVybmluZyBhIGNsZWFuIHBsb3QtcmVhZHkgZGYNCnByZXBfcGxvdF9kYXQgPC0gZnVuY3Rpb24oZGF0LCB4X3Zhcikgew0KICBkYXQgfD4NCiAgICBtdXRhdGUoDQogICAgICB4X3JhdyA9IHN0cl90cmltKHRvbG93ZXIoLmRhdGFbW3hfdmFyXV0pKSwNCiAgICAgIHhfcGxvdCA9IG5hX2lmKHhfcmF3LCAiIikNCiAgICApIHw+DQogICAgZmlsdGVyKCFpcy5uYSh4X3Bsb3QpLA0KICAgICAgICAgICAheF9wbG90ICVpbiUgYygibmEiLCAibi9hIiwgIm4uYS4iLCAibmFuIikpIHw+DQogICAgZ3JvdXBfYnkoeF9wbG90LCBCcm9hZGVyKSB8Pg0KICAgIG11dGF0ZSgNCiAgICAgIHExICA9IHF1YW50aWxlKFNOUF9sb2csIDAuMjUsIG5hLnJtID0gVFJVRSksDQogICAgICBxMyAgPSBxdWFudGlsZShTTlBfbG9nLCAwLjc1LCBuYS5ybSA9IFRSVUUpLA0KICAgICAgaXFyID0gcTMgLSBxMSwNCiAgICAgIGxvICA9IHExIC0gMS41ICogaXFyLA0KICAgICAgaGkgID0gcTMgKyAxLjUgKiBpcXINCiAgICApIHw+DQogICAgZmlsdGVyKGJldHdlZW4oU05QX2xvZywgbG8sIGhpKSkgfD4NCiAgICBmaWx0ZXIobigpID4gNSkgfD4NCiAgICB1bmdyb3VwKCkNCn0NCg0KIyBIZWxwZXI6IG92ZXJhbGwgbWVhbiArIDk1JSBDSSAob24gbG9nIHNjYWxlKSBieSB4X3Bsb3QNCm92ZXJhbGxfY2kgPC0gZnVuY3Rpb24oZGF0KSB7DQogIGRhdCB8Pg0KICAgIGdyb3VwX2J5KHhfcGxvdCkgfD4NCiAgICBzdW1tYXJpc2UoDQogICAgICBtZWFuID0gbWVhbihTTlBfbG9nLCBuYS5ybSA9IFRSVUUpLA0KICAgICAgc2QgICA9IHNkKFNOUF9sb2csIG5hLnJtID0gVFJVRSksDQogICAgICBuICAgID0gbigpLA0KICAgICAgc2UgICA9IHNkIC8gc3FydChuKSwNCiAgICAgIGx3ciAgPSBtZWFuIC0gMS45NiAqIHNlLA0KICAgICAgdXByICA9IG1lYW4gKyAxLjk2ICogc2UsDQogICAgICAuZ3JvdXBzID0gImRyb3AiDQogICAgKQ0KfQ0KDQojIEhlbHBlcjogYnVpbGQgdGhlIGdncGxvdCAoYm94ICsgaml0dGVyICsgb3ZlcmFsbCBtZWFuL0NJKQ0KbWFrZV9wbG90IDwtIGZ1bmN0aW9uKGRhdCwgb3ZlcmFsbCwgdGl0bGVfbGV0dGVyLCB4bGFiX3dyYXAgPSAxMCkgew0KICBnZ3Bsb3QoZGF0LCBhZXMoeCA9IHhfcGxvdCwgeSA9IFNOUF9sb2csIGNvbG9yID0gQnJvYWRlcikpICsNCiAgICBnZW9tX2JveHBsb3QoDQogICAgICBwb3NpdGlvbiA9IHBvc2l0aW9uX2RvZGdlKHdpZHRoID0gMC43NSksDQogICAgICB3aWR0aCA9IDAuNiwNCiAgICAgIGxpbmV3aWR0aCA9IDAuMywNCiAgICAgIG91dGxpZXIuc2hhcGUgPSBOQQ0KICAgICkgKw0KICAgIGdlb21faml0dGVyKA0KICAgICAgcG9zaXRpb24gPSBwb3NpdGlvbl9qaXR0ZXJkb2RnZSgNCiAgICAgICAgaml0dGVyLndpZHRoID0gMC4xNSwNCiAgICAgICAgZG9kZ2Uud2lkdGggPSAwLjc1DQogICAgICApLA0KICAgICAgc2l6ZSA9IDAuMywNCiAgICAgIGFscGhhID0gMC43DQogICAgKSArDQogICAgZ2VvbV9lcnJvcmJhcigNCiAgICAgIGRhdGEgPSBvdmVyYWxsLA0KICAgICAgYWVzKHggPSB4X3Bsb3QsIHltaW4gPSBsd3IsIHltYXggPSB1cHIpLA0KICAgICAgd2lkdGggPSAwLjIsDQogICAgICBpbmhlcml0LmFlcyA9IEZBTFNFLA0KICAgICAgY29sb3IgPSAiYmxhY2siDQogICAgKSArDQogICAgZ2VvbV9wb2ludCgNCiAgICAgIGRhdGEgPSBvdmVyYWxsLA0KICAgICAgYWVzKHggPSB4X3Bsb3QsIHkgPSBtZWFuKSwNCiAgICAgIGluaGVyaXQuYWVzID0gRkFMU0UsDQogICAgICBjb2xvciA9ICJibGFjayIsDQogICAgICBzaXplID0gMS41DQogICAgKSArDQogICAgc2NhbGVfeF9kaXNjcmV0ZShsYWJlbHMgPSBmdW5jdGlvbih4KSBzdHJfd3JhcCh4LCB3aWR0aCA9IHhsYWJfd3JhcCkpICsNCiAgICBzY2FsZV9jb2xvcl9tYW51YWwodmFsdWVzID0gYnJvYWRlcl9wYWwsIGJyZWFrcyA9IGJyb2FkZXJfbGV2ZWxzLCBkcm9wID0gRkFMU0UpICsNCiAgICB0aGVtZV9taW5pbWFsKDEwKSArDQogICAgbGFicyh0aXRsZSA9IHRpdGxlX2xldHRlciwgeCA9ICIiLCB5ID0gIiIpICsNCiAgICB0aGVtZSgNCiAgICAgIGF4aXMudGV4dC54ID0gZWxlbWVudF90ZXh0KHNpemUgPSA4KSwNCiAgICAgIGF4aXMudGV4dC55ID0gZWxlbWVudF90ZXh0KHNpemUgPSA4KSwNCiAgICAgIGxlZ2VuZC5wb3NpdGlvbiA9ICJub25lIg0KICAgICkNCn0NCmBgYA0KDQojIyBhLCBiLCBhbmQgYzoNCg0KYGBge3J9DQojIFBsb3QgQTogdmFyaWFibGUuZ3JvdXBpbmcNCnBsb3RfYV9kYXQgPC0gcHJlcF9wbG90X2RhdChiYXNlX2RhdCwgInZhcmlhYmxlLmdyb3VwaW5nIikNCnBsb3RfYV9kYXQgPC0gcGxvdF9hX2RhdCB8PiBtdXRhdGUoQnJvYWRlciA9IGZhY3RvcihCcm9hZGVyLCBsZXZlbHMgPSBicm9hZGVyX2xldmVscykpDQoNCiMgWW91ciBkZXNpcmVkIG9yZGVyIGZvciBBIChlZGl0IGFzIG5lZWRlZCkNCnZhcl9vcmRlcl9hIDwtIGMoDQogICJ0ZW1wLiBtZWFuIiwgInRlbXAuIGV4dC4iLCAidGVtcC4gdmFyLiIsDQogICJwcmVjaXAuIGFubnVhbCIsICJwcmVjaXAuIHF1LiIsICJwcmVjaXAuIGV4dC4iLCAicHJlY2lwLiB2YXIuIiwNCiAgInByb2QuIG1lYW4iLCAicHJvZC4gZXh0LiIsDQogICJ3YXRlciBjb25kLiBtZWFuIiwgImhhYi4gY29uZC4iLA0KICAidG9wby4vaHlkcm8uIiwgInNwYXRpYWwiDQopDQoNCnBsb3RfYV9kYXQgPC0gcGxvdF9hX2RhdCB8Pg0KICBtdXRhdGUoeF9wbG90ID0gZmFjdG9yKHhfcGxvdCwgbGV2ZWxzID0gdmFyX29yZGVyX2EpKQ0KDQojIEZJTFRFUiBUT1BPIEFORCBTUEFDRSBzbyBvdGhlciBjYXRlZ29yaWVzIGNhbiBiZSBtb3JlIGVhc2lseSB2aXN1YWxpemVkLiANCiMgVGhlc2UgY2F0ZWdvcmllcyBoYXZlIGxvdyBudW1iZXJzIG9mIG9ic2VydmF0aW9ucyBhbmQgb25seSAxIHRheG9ub21pYyBncm91cA0KcGxvdF9hX2RhdCA8LSBwbG90X2FfZGF0IHw+DQogIGZpbHRlcigheF9wbG90ICVpbiUgYygidG9wby4vaHlkcm8uIiwgInNwYXRpYWwiKSkNCg0Kb3ZlcmFsbF9hIDwtIG92ZXJhbGxfY2kocGxvdF9hX2RhdCkNCmEgPC0gbWFrZV9wbG90KHBsb3RfYV9kYXQsIG92ZXJhbGxfYSwgImEiKSArIGd1aWRlcyhjb2xvciA9ICJub25lIikNCg0KYQ0KDQojIFBsb3QgQjogdmFyaWFibGVfdHlwZQ0KcGxvdF9iX2RhdCA8LSBwcmVwX3Bsb3RfZGF0KGJhc2VfZGF0LCAidmFyaWFibGVfdHlwZSIpDQpwbG90X2JfZGF0IDwtIHBsb3RfYl9kYXQgfD4gbXV0YXRlKEJyb2FkZXIgPSBmYWN0b3IoQnJvYWRlciwgbGV2ZWxzID0gYnJvYWRlcl9sZXZlbHMpKQ0KDQojIFlvdXIgZGVzaXJlZCBvcmRlciBmb3IgQiAoZWRpdCBhcyBuZWVkZWQpDQp2YXJfb3JkZXJfYiA8LSBjKCJ0ZW1wLiIsICJwcmVjaXAuIiwgIndhdGVyIGNvbmQuIiwgInByb2QuIiwgInNpdGUiKQ0KDQpwbG90X2JfZGF0IDwtIHBsb3RfYl9kYXQgfD4NCiAgbXV0YXRlKHhfcGxvdCA9IGZhY3Rvcih4X3Bsb3QsIGxldmVscyA9IHZhcl9vcmRlcl9iKSkNCg0Kb3ZlcmFsbF9iIDwtIG92ZXJhbGxfY2kocGxvdF9iX2RhdCkNCmIgPC0gbWFrZV9wbG90KHBsb3RfYl9kYXQsIG92ZXJhbGxfYiwgImIiKSArDQogIHRoZW1lKGxlZ2VuZC5wb3NpdGlvbiA9ICJyaWdodCIpICsgbGFicyggeSA9ICJsb2cxMChTTlAgZGV0ZWN0aW9uIHJhdGUpIikNCg0KYg0KDQojIFBsb3QgQzogbWVhc3VyZW1lbnRfc3VtbWFyeV90eXBlDQoNCnBsb3RfY19kYXQgPC0gcHJlcF9wbG90X2RhdChiYXNlX2RhdCwgIm1lYXN1cmVtZW50X3N1bW1hcnlfdHlwZSIpDQpwbG90X2NfZGF0IDwtIHBsb3RfY19kYXQgfD4gbXV0YXRlKEJyb2FkZXIgPSBmYWN0b3IoQnJvYWRlciwgbGV2ZWxzID0gYnJvYWRlcl9sZXZlbHMpKQ0KDQojIEZpbGwgaW4gbGF0ZXI6DQp2YXJfb3JkZXJfYyA8LSBjKA0KICAibWVhbiIsICJleHRyZW1lIiwgInZhcmlhdGlvbiIsICJyYXciDQopDQpwbG90X2NfZGF0IDwtIHBsb3RfY19kYXQgfD4NCiAgIG11dGF0ZSh4X3Bsb3QgPSBmYWN0b3IoeF9wbG90LCBsZXZlbHMgPSB2YXJfb3JkZXJfYykpDQoNCm92ZXJhbGxfYyA8LSBvdmVyYWxsX2NpKHBsb3RfY19kYXQpDQpjIDwtIG1ha2VfcGxvdChwbG90X2NfZGF0LCBvdmVyYWxsX2MsICJjIikgKyBndWlkZXMoY29sb3IgPSAibm9uZSIpICsNCiAgbGFicyh4ID0gIlZhcmlhYmxlIGNhdGVnb3J5IikNCg0KYw0KDQpgYGANCg0KU2F2ZQ0KDQpgYGB7cn0NCmNvbWJpbmVkIDwtIChhIC8gYiAvIGMpICsNCiAgcGxvdF9sYXlvdXQoZ3VpZGVzID0gImNvbGxlY3QiLCBoZWlnaHRzID0gYygxLCAxLCAxKSkgJg0KICB0aGVtZSgNCiAgICBsZWdlbmQucG9zaXRpb24gPSAiYm90dG9tIiwNCiAgICBsZWdlbmQudGl0bGUgPSBlbGVtZW50X2JsYW5rKCksDQogICAgcGxvdC5tYXJnaW4gPSBtYXJnaW4oMCwgMCwgMCwgMCkNCiAgKQ0KDQpjb21iaW5lZA0KDQpnZ3NhdmUoIkZpZzJhLWMucGRmIiwNCiAgICAgICBwbG90ID0gY29tYmluZWQsDQogICAgICAgZGV2aWNlID0gY2Fpcm9fcGRmLCAgICMgYmVzdCB0ZXh0IHJlbmRlcmluZw0KICAgICAgIHdpZHRoID0gMTc0LCBoZWlnaHQgPSAyMzAsIHVuaXRzID0gIm1tIikNCmBgYA0K
